# The comparative immunology of wild and laboratory mice, *Mus musculus domesticus*

**DOI:** 10.1038/ncomms14811

**Published:** 2017-05-03

**Authors:** Stephen Abolins, Elizabeth C. King, Luke Lazarou, Laura Weldon, Louise Hughes, Paul Drescher, John G. Raynes, Julius C. R. Hafalla, Mark E. Viney, Eleanor M. Riley

**Affiliations:** 1 School of Biological Sciences, University of Bristol, Tyndall Avenue, Bristol BS8 1TQ, UK; 2 Department of Immunology and Infection, London School of Hygiene and Tropical Medicine, Keppel Street, London WC1E 7HT, UK

## Abstract

The laboratory mouse is the workhorse of immunology, used as a model of mammalian immune function, but how well immune responses of laboratory mice reflect those of free-living animals is unknown. Here we comprehensively characterize serological, cellular and functional immune parameters of wild mice and compare them with laboratory mice, finding that wild mouse cellular immune systems are, comparatively, in a highly activated (primed) state. Associations between immune parameters and infection suggest that high level pathogen exposure drives this activation. Moreover, wild mice have a population of highly activated myeloid cells not present in laboratory mice. By contrast, *in vitro* cytokine responses to pathogen-associated ligands are generally lower in cells from wild mice, probably reflecting the importance of maintaining immune homeostasis in the face of intense antigenic challenge in the wild. These data provide a comprehensive basis for validating (or not) laboratory mice as a useful and relevant immunological model system.

## Abstract (web summary)

Laboratory mice are the cornerstone of immunology but how well they represent wild mice is not clear. Here the authors compare and contrast various immune parameters between wild-caught mice and laboratory (C57BL/6) mice and identify a previously unknown myeloid cell population specific to wild mice.

Most of our understanding of the mammalian immune system comes from detailed studies of inbred, laboratory-adapted strains of the house mouse, *Mus musculus domesticus*, but whether such responses are indicative of those of free-living, outbred populations is unknown. Laboratory mice have been genetically isolated from their free-living relatives for more than 80 years such that laboratory strains capture only a small part of the genetic variation present in wild populations^1^,^2^,^3^. The genome of the laboratory mouse is a mosaic of the *M. musculus domesticus* and *Mus musculus musculus* genomes^4^. Laboratory mouse strains are also mostly genetically homozygous often resulting in phenotypes caused by recessive alleles. Indeed, major differences exist among inbred mouse strains in immune phenotype and function, and resistance or susceptibility to infectious or inflammatory diseases. Many of these traits have been mapped to specific loss of function mutations in genes that affect the immune response^5^.

The different genetic heritage of wild and laboratory mice is obvious in other ways given that laboratory mice are larger and heavier than, and differ in coat colour from, wild *M. musculus domesticus*. In adaptation to the laboratory, mice have been selected for rapid growth, early maturation, high fecundity and docility^6^ and inadvertent selection for immunological traits is almost certain. Equally important for immune function, laboratory mice typically live in highly controlled and optimized environments, have unlimited access to food and are kept free of pathogens; the increasing trend towards housing animals in individually ventilated cages further reduces exposure to environmental antigens. By contrast, wild mice are continually exposed to environmental antigens, are typically infected by numerous microparasites and macroparasites, and face competition for resources (for example, food, mates, safe nesting places). Wild mouse populations are subject to continual selection in this very different antigenic and physical environment, where immune responses make an important contribution to their fitness.

Given these substantial differences between wild and laboratory animals and their respective environments, differences between their baseline immune parameters, immune responses to model antigens and functional immune competence are expected. Understanding immune phenotype and function in wild mice is essential for understanding immune responses of genetically diverse, free-living populations, including humans^7^,^8^. Comparison of the immune function of wild and laboratory mice is also required to reveal both the utility and the limitations of laboratory mice as broadly applicable and relevant immunological models.

Although immune function might be assumed to differ between wild and laboratory mice, this assumption is based on remarkably little empirical evidence; there are only four published reports of the immune function of wild *M. musculus domesticus* (refs 9, 10, 11, 12). It is remarkable that mice—the central model used in immunology—are so immunologically understudied in the wild. In these studies, wild caught mice co-housed with laboratory mice and immunized with either sheep red blood cells^9^ or keyhole limpet haemocyanin^10^ developed higher anti-sheep red blood cells and anti-keyhole limpet haemocyanin antibody responses (greater lytic activity; and higher concentration and avidity, respectively) than laboratory mice. Small scale, *ex vivo* flow cytometric studies report higher proportions of activated CD4^+^ T cells, B cells, macrophages and dendritic cells in spleens of wild mice compared to laboratory mice^10^, and skewing of the natural killer (NK) cell population to a supposedly less mature phenotype but with higher resting levels of NK cell activation in wild mice compared with laboratory mice^11^. A small-scale comparison of wild and pet mice with laboratory mice found that the non-laboratory mice had more antigen-experienced CD8^+^ T cells than laboratory mice, commensurate with living in unsterile conditions^12^. Although these studies support the idea that wild and laboratory mice differ immunologically, the lack of an extensive, immune system-wide analysis of populations of wild mice tempers this conclusion. It therefore remains the case that the immune responses of wild mice are essentially unknown and thus that the validity of laboratory mice as a model immunological system is uncertain.

We have therefore undertaken a detailed phenotypic and functional analysis of the immune systems of 460 wild mice (*M. musculus domesticus*) from 12 sites in the southern UK, comprising ten farms in the Bristol region, the island of Skokholm (Pembrokeshire, south west Wales) and the London Underground (Fig. 1a). The entire data set comprises (1) serum concentrations of immunoglobulins (IgG and IgE) and acute phase proteins (serum amyloid P and haptoglobin); (2) faecal IgA concentrations; (3) *ex vivo* flow cytometric analysis of splenocytes (proportions, absolute numbers and activation status of T cells, B cells, regulatory T [T_reg_] cells, NK cells, dendritic cells and myeloid cells); (4) multiplex bead array analysis of cytokine production after *in vitro* stimulation of splenocytes with pathogen-associated molecular patterns (PAMPs), including CpG, peptidoglycan (PG) and lipopolysaccharide (LPS) and mitogenic stimulation with anti-CD3 and anti-CD28 antibodies.

## Results

### A community resource

**Figure 1:**
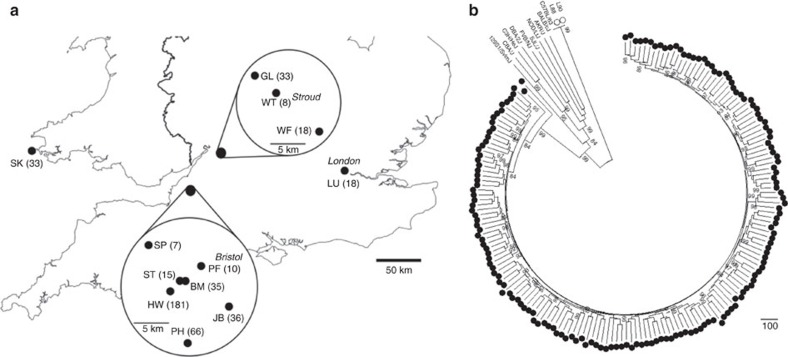
Wild mice sampled for immune characterization. (**a**) The wild mouse sampling sites, the site codes (and showing Bristol, Stroud and London; Supplementary Table 1) and the number of mice sampled at each site. (**b**) A neighbour-joining tree of the wild mice (HW) (●) and laboratory strains of mice (○). The scale is the number of nucleotide differences among individuals. Bootstrapping values are displayed for branches that have >80% support after 1,000 runs. L88 and L90 are two C57BL/6 mice that we genotyped (Supplementary Data 3) and compared to published data^31^ for this strain.

The complete immunological data set of 460 wild mice is provided as a community resource (Supplementary Data 1). From this we compare in detail a subset of 181 wild mice (100 male, 81 female) from a single site (site HW, Fig. 1a, Supplementary Table 1) with 64 laboratory-reared, pathogen-free C57BL/6 (24 male, 40 female) mice. The results of this comparison are shown in Tables 1,2 and Supplementary Table 2, the latter being too large to fit within the main text of the article.

### Wild mice are immunologically different from laboratory mice

Serological and morphometric parameters for the wild (HW) and laboratory (C57/BL6) mice are summarized in Table 1. The wild mice were much smaller than the laboratory mice (weighing only half as much) and among the wild mice, age, body length and mass were all highly correlated (length and mass, Pearson correlations (two-tailed) *r*=0.79; age and mass, *r*≥0.77; age and length, *r*=0.58, *P*<0.001, *n*>80 for male and female mice separately) (Supplementary Data 2). The wild mice had a median age of 6.6 weeks (range 1–39.5) and many immune parameters correlated with age and size, likely due to cumulative exposure to infection (Supplementary Data 2). From 62 immunological measures most (57 measures) differed between wild and laboratory mice (Table 1, Table 2, Supplementary Table 2). Among the wild mice there were very few (6 of 62 measures) significant immunological differences between male and female mice, while the laboratory mice were more (18 of 62 measures) immunologically sexually dimorphic (Table 1, Table 2, Supplementary Table 2).

Multilocus genotyping shows that the HW wild mice are an unstructured, genetically diverse population (Fig. 1b, Supplementary Data 3). The wild mice are genetically distinct from ten laboratory mouse strains, and the laboratory strains are more genetically diverse than are the wild mice. We suggest that this genetic relationship between the wild and laboratory mice is explained by the mosaicisim of laboratory mouse genomes^4^, by the fact that laboratory mice have been deliberately separated from each other for many generations, and by the fact that laboratory strains are largely homozygous.

### Wild mice carry a substantial burden of infection

We screened the wild mice for evidence of infection with viruses and *Mycoplasma pulmonis*, and for evidence of ectoparasite and intestinal nematode infection; suppliers confirmed the laboratory mice to be infection free. The seroprevalence of the different microbial infections ranged from 22% for minute virus to 92% for parvovirus (*n*=153 for both analyses; Supplementary Table 3). Wild mice were commonly infected with the Oxyurid nematode *Syphacia* spp. (prevalence 91%) and with the mite *Myocoptes musculinus* (prevalence 82%) (*n*=181 in both cases). Infection of wild mice was very common: all wild mice had been infected with at least one pathogen and only 5% (8 of 153) were seronegative for all the viruses and *M. pulmonis*. There was no effect of sex on the intensity or prevalence of infection (Supplementary Table 3).

### Wild mice have very high concentrations of serum proteins

**Figure 2:**
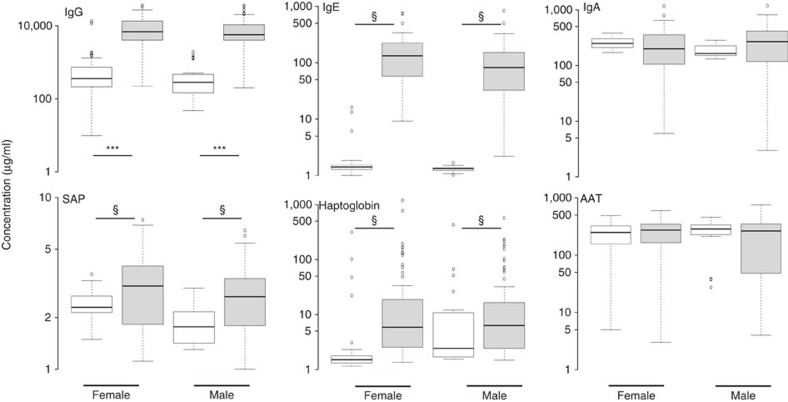
Immunoglobulins and serum proteins. Immunoglobulin G, E and A, and SAP, haptoglobin, and AAT serum proteins concentrations of wild (shaded) and laboratory (unshaded) mice are shown on a log_10_ scale. The box centres are medians, and box limits the 25th and 75th percentiles, the whiskers 1.5 times the interquartile range, and outliers are represented by dots. Asterisks denote significant differences as ***P<0.001 (Mann–Whitney *U* test; Table 1), and § denotes that there are additional sex effects detailed in Table 1. Sample sizes are shown in Table 1 and Supplementary Data 1.

In wild mice, serum concentrations of IgG and IgE were 20- and 200-fold higher, respectively, in wild mice than in the laboratory mice (Fig. 2). Among wild mice, IgE concentrations were significantly higher among females than among males (Table 1). By contrast, faecal IgA concentrations did not differ significantly between wild and laboratory mice (Fig. 2, Table 1). Wild mice also had significantly higher serum concentrations of the acute phase proteins, serum amyloid P component (SAP) and haptoglobin than laboratory mice (Fig. 2, Table 1). These differences were not due to higher total serum protein concentrations in wild mice since concentrations of alpha-1 antitrypsin (AAT)—a stable component of normal serum—did not differ between wild and laboratory mice (Fig. 2, Table 1).

Wild mice were more heterogeneous in their concentrations of immunoglobulins and acute phase proteins compared with laboratory mice (Fig. 2, Table 1, Supplementary Table 4). Although baseline SAP concentrations are partially genetically determined^13^, the significant correlation between SAP and haptoglobin concentrations (Pearson correlations (two-tailed) *r*=0.41, *P*<0.0001, *r*=0.33, *P*=0.004 for 96 males and 77 females, respectively; Supplementary Data 2) suggests that inflammation and/or infection are the probable drivers of this heterogeneity. Among wild mice, the serum concentrations of IgG and IgE were significantly, positively correlated with age (Pearson correlation (two-tailed) *r*>0.2, *P*<0.05, *n*≥79; Supplementary Data 2) likely reflecting cumulative exposure to infection. This can be seen explicitly for IgE concentrations which were significantly positively correlated with the number of microbial infections in male wild mice (Pearson correlation (two-tailed) *r*=0.23, *P*=0.036, *n*=80; Supplementary Data 2). In female wild mice, faecal IgA concentration was highly correlated with the number of microbial infections and with the number of mites (microbial infections Pearson correlations (two-tailed) *r*=0.58, *P*<0.0001, *n*=35; number of mites *r*=−0.380, *P*=0.01, *n*=45; Supplementary Data 2).

### Wild mouse splenocytes differ from those of laboratory mice

Spleens of wild mice were much smaller (approximately one third the mass) than those of laboratory mice and contained significantly fewer (approximately one fifth the number) viable mononuclear leucocytes (Table 1). More surprisingly, the spleens of wild mice were significantly proportionately smaller (that is, when compared with body mass) than those of laboratory mice (Table 1).

**Figure 3:**
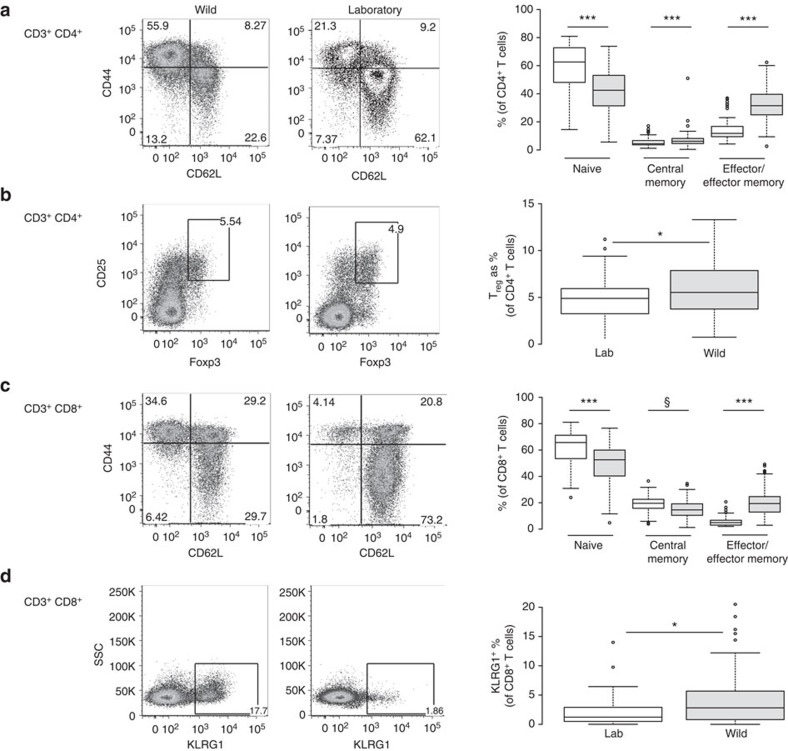
Splenic T-cell populations. The flow cytometry gating strategy and proportions of subsets of CD3^+^ T cells in wild (shaded) and laboratory (unshaded) mice for (**a**) CD4^+^ cells, (**b**) CD4^+^ T_reg_ cells, (**c**) CD8^+^ cells and their maturation state and (**d**) terminally differentiated CD8^+^ cells. CD4^+^ and CD8^+^ effector/effector memory cells are defined as CD62L^−^ CD44^hi^ and central memory cells are CD62L^+^ CD44^hi^. The box centres are medians, and box limits the 25th and 75th percentiles, the whiskers 1.5 times the interquartile range and outliers are represented by dots. Asterisks denote significant differences as *P<0.05, **P<0.01, ***P<0.001 (Mann–Whitney *U* test; Supplementary Table 2), and § denotes that there are additional sex effects detailed in Supplementary Table 2. The gating strategy for CD3^+^ lymphocytes is shown in Supplementary Fig. 1. Sample sizes are shown in Supplementary Table 2 and Supplementary Data 1.

**Figure 4:**
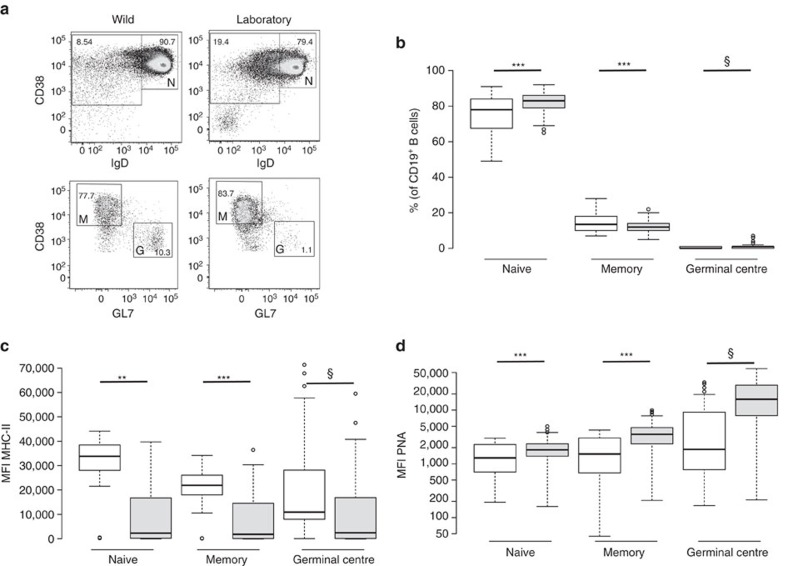
Splenic B-cell populations. (**a**) The flow cytometry gating strategy to characterize CD19^+^ B cells as either naïve (N), memory (M) or germinal centre (G) B cells in wild and laboratory mice, and (**b**) the proportions of these three subpopulations, (**c**) their expression of MHC class II and (**d**) binding of PNA, with the latter shown on a log_10_ scale. Mice are wild (shaded) and laboratory (unshaded). The box centres are medians, and box limits the 25th and 75th percentiles, the whiskers 1.5 times the interquartile range and outliers are represented by dots. Asterisks denote significant differences as **P<0.01, ***P<0.001 (Mann–Whitney *U* test; Supplementary Table 2), and § denotes that there are additional sex effects detailed in Supplementary Table 2. Sample sizes are shown in Supplementary Table 2 and Supplementary Data 1. The gating strategy for CD19^+^ lymphocytes is shown in Supplementary Fig. 1.

**Figure 5:**
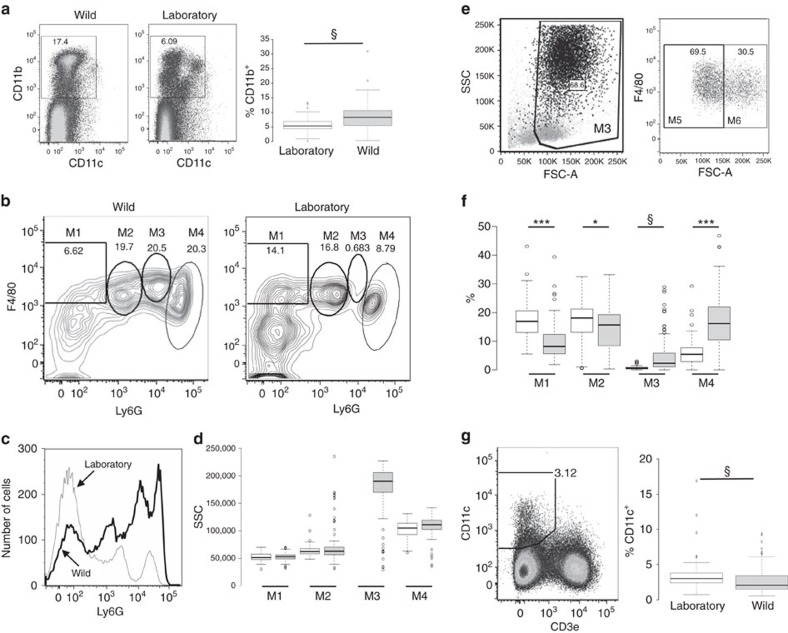
Myeloid cells. (**a**) The flow cytometry gating strategy to identify CD11b^+^ CD11c^−^ myeloid cells and the proportion of myeloid cells among splenic leukocytes in wild (shaded) and laboratory (unshaded) mice, (**b**) gating myeloid cells on F4/80 and Ly6G expression to define M1 (tissue resident macrophages), M2 (monocytes), M3 (hypergranulocytic myeloid cells, HGMC) and M4 (polymorphonuclear leukocytes, PMN) subsets, (**c**) Ly6G expression confirming the presence of three cell populations in laboratory mice and four populations in wild mice, (**d**) side scatter characteristics of the M1–M4 populations in wild (shaded, *n*≥115) and laboratory (unshaded *n*≥57) mice; note that too few cells were present in the M3 gate in laboratory mice to accurately determine a side scatter statistic, (**e**) scatter characteristics of M3 (left) and M4 (right) cells, revealing a low forward scatter neutrophil population (M5) and a high forward scatter myeloid derived suppressor cell population (M6) among the M4 cells, (**f**) proportions of M1, M2, M3 and M4 subpopulations among the myeloid cell population in wild (shaded) and laboratory (unshaded) mice, and (**g**) gating of CD11c^+^ dendritic cells and their proportions among splenocytes in wild and laboratory mice. For the box plots, box centres are medians, and box limits the 25th and 75th percentiles, the whiskers 1.5 times the interquartile range and outliers are represented by dots. Asterisks denote significant differences as *P<0.05, ***P<0.001 (Mann–Whitney *U* test; Supplementary Table 2), and § denotes that there are additional sex effects detailed in Supplementary Table 2. Sample sizes are shown in Supplementary Table 2 and Supplementary Data 1.

**Figure 6:**
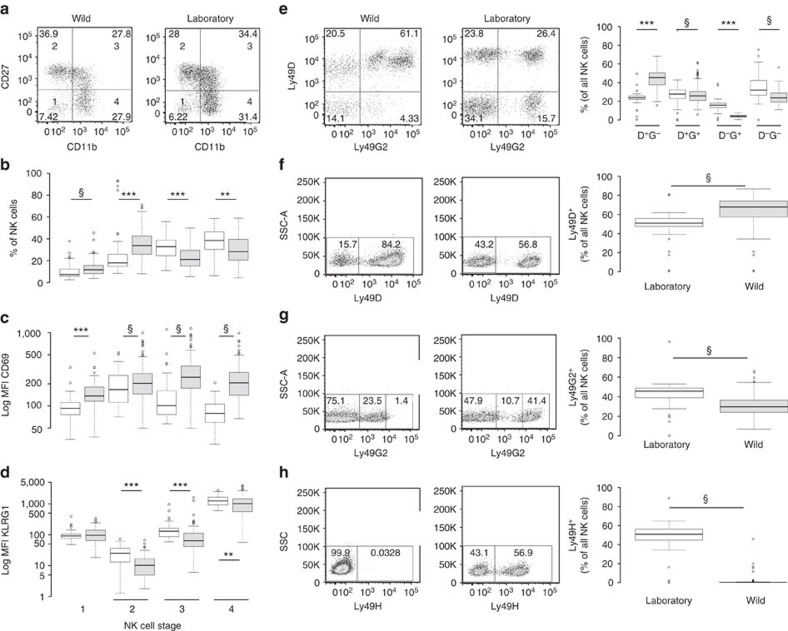
Splenic NK cells and Ly49 expression. (**a**) The flow cytometry gating strategy using expression of CD27 and CD11b to classify NKp46^+^ CD3^−^ splenic NK cells of wild and laboratory mice into stages 1–4 of maturity, (**b**) the proportions of NK cells at each such stage, in wild (shaded) and laboratory (unshaded) mice, and expression of (**c**) CD69 and (**d**) KLRG1 by each subset (Table 2). Also shown are (**e**–**h**) the gating strategies for Ly49 receptors and the proportions of NK cells expressing, (**e**) different combinations of Ly49D and Ly49G2, (**f**) Ly49D, (**g**) Ly49G2 (Ly49G2^+^ cells gated into Ly49G2^−^, Ly49G2^low^ and Ly49G2^high^ cells) and (**h**) Ly49H. Wild mice are shown as shaded box plots, laboratory mice as unshaded. The box centres are medians, and box limits the 25th and 75th percentiles, the whiskers 1.5 times the interquartile range and outliers are represented by dots and some axes are on a log_10_ scale. Asterisks denote significant differences as **P<0.01, ***P<0.001 (Mann–Whitney *U* test; Table 2), and § denotes that there are additional sex effects detailed in Table 2. The gating strategy for NKp46^+^ CD3^−^ lymphocytes is shown in Supplementary Fig. 1. Sample sizes are shown in Table 2 and Supplementary Data 1.

*Ex vivo* flow cytometric quantification and characterization of spleen cell populations (Figs 3, 4, 5, 6, Supplementary Fig. 1) revealed that the wild mice had lower absolute numbers of T cells, B cells, NK cells, dendritic cells, macrophages and neutrophils than laboratory mice, consistent with their lower absolute number of splenic mononuclear cells (Supplementary Data 1). But, proportionately, wild mouse spleens had significantly more T cells, a higher T:B cell ratio and more CD11b^+^ myeloid cells, but fewer NK cells and dendritic cells, than laboratory mice (Supplementary Table 2); the ratio of CD4^+^: CD8^+^ T cells was also significantly higher in wild mice than in laboratory mice. These differences are consistent with accumulation of T helper cells and phagocytic cells in the spleens of wild mice in response to systemic infections.

The status of CD4^+^ and CD8^+^ T cells was markedly different between wild and laboratory mice. For CD4^+^ T cells significantly greater proportions were effector/effector memory (CD62L^−^ CD44^hi^) and central memory (CD62L^+^ CD44^hi^) cells (and so proportionately fewer were naïve, CD62L^+^ CD44^low^), in wild mice compared to laboratory mice (Supplementary Table 2, Fig. 3a). Although proportions of CD4^+^ T cells that were Foxp3^+^ CD25^+^ T_reg_ cells were marginally higher among wild than laboratory mice (Supplementary Table 2, Fig. 3b), this was insufficient to offset the much larger proportion of effector CD4^+^ T cells such that ratios of effector CD4^+^ T cells to T_regs_ were significantly higher among wild than laboratory mice (Supplementary Table 2).

Similarly, for CD8^+^ T cells wild mice had a significantly higher proportion of effector/effector memory (CD62L^−^ CD44^hi^) and terminally differentiated (KLRG1^+^) cells than did laboratory mice (and so significantly lower proportions of naïve) (Fig. 3c,d). Wild mice also had proportionately fewer central memory (CD62L^+^ CD44^hi^) CD8^+^ T cells than laboratory mice; this difference was due in part to the low frequency of these cells in wild male mice (Supplementary Table 2), but may also reflect the relative distribution of antigen-experienced CD8^+^ T cells between the memory and effector subsets. Again, the ratio of effector/effector memory CD8^+^ T cells to T_regs_ was significantly higher in wild than laboratory mice (Supplementary Table 2).

Consistent with the idea that frequent or persistent pathogen challenge drives expansion of antigen-experienced CD4^+^ and CD8^+^ T-cell subsets in wild mice, there were significant positive correlations between the proportions of effector CD4^+^ and CD8^+^ T cells and age among female wild mice (Pearson correlations (two-tailed) age and effector CD4^+^ *r*=0.62, *P*<0.0001, *n*=51; age and effector CD8^+^ *r*=0.49, *P*<0.0001, *n*=50; Supplementary Data 2). Interestingly, these parameters were not strongly correlated with age in male wild mice (Pearson correlation (two-tailed) *r*<0.1, *P*>0.05, *n*=66) pointing to different immunological strategies of male and female mice in the wild.

In contrast to the highly primed/effector status of splenic T cells, CD19^+^ B lymphocytes of wild mice predominantly had a naïve phenotype. We categorized splenic CD19^+^ B lymphocytes as naïve (CD38^+^ IgD^+^), memory (CD38^+^ IgD^−^ GL7^−^) or germinal centre (CD38^lo^ IgD^−^ GL7^hi^) cells^14^, and identified recently activated, antigen-experienced cells by their MHC class II expression and binding of peanut agglutinin (PNA; indicating expression of the PNA receptor, PNA-R)^15^ (Fig. 4a). Despite their very high serum immunoglobulin concentrations, spleens of wild mice contained significantly higher proportions of naïve B cells (and, reciprocally, significantly lower proportions of memory B cells) than the laboratory mice (Fig. 4b,c). This initially counter-intuitive observation probably reflects reallocation of antigen-experienced B cells from the spleen to the bone marrow, to other lymphoid tissues, or to sites of infection, together with continual repopulation of the spleen by naïve, bone marrow-derived B cells. Wild mice had proportionately more germinal centre B cells in their spleens than did laboratory mice and PNA binding was comparatively higher on all B-cell subsets in wild mice, consistent with recent activation^15^ (Fig. 4d, Supplementary Table 2). Together these results point to high turnover of activated CD19^+^ B cells in wild mouse spleens.

### Wild mice have a hitherto unknown myeloid cell population

We next identified myeloid cells as CD11b^+^ CD11c^−^ (Fig. 5a) and analysed their expression of F4/80 and Ly6G, revealing four subpopulations of F4/80^+^ cells, denoted M1–M4 (Fig. 5b–d). These include F4/80^+^ Ly6G^−^ (M1) tissue resident macrophages, F4/80^+^ Ly6G^low^ (M2) monocytes/red pulp macrophages and F4/80^+/−^Ly6G^high^ (M4) polymorphonuclear cells (PMN). The M4 PMN population could be further divided into neutrophils and myeloid-derived suppressor cells based on their forward and side scatter characteristics (Fig. 5e). Importantly, in wild, but not in laboratory, mice, we identified an additional population of F4/80^+^ cells expressing levels of Ly6G that are intermediate between monocyte/macrophages and PMN (M3). As far as we are aware, this is a novel population of cells not previously described, which we have termed hyper-granulocytic myeloid cells (HGMC) on the basis of their forward and side scatter characteristics (Fig. 5c–e). Although there are some slight differences in levels of Ly6G expression between the M2 and M3/M4 populations in wild and laboratory mice (Fig. 5c) back gating of each population on CD11b, CD11c, and forward and side scatter confirmed that the M2 populations in wild and laboratory mice are otherwise identical and that the Ly6G^high^ population in laboratory mice is equivalent to the M4 population in wild mice (Fig. 5d). Comparison of the side scatter for each population also confirms that the high side scatter, hypergranulocytic M3 population is indeed seen only in wild mice (Fig. 5f). The functional significance of these cells is as yet unknown, but their discovery emphasizes that study of laboratory mice does not necessarily reveal the full armamentarium of the immune system.

Wild mice not only had proportionally more CD11b^+^ CD11c^−^ myeloid cells in their spleens than did laboratory mice but, within the myeloid population, PMN and HGMC were enriched at the expense of macrophages and monocytes (Fig. 5a,f, Supplementary Table 2). The expansion and/or accumulation of neutrophils and HGMC in spleens of wild mice is consistent with recent or current exposure to infection in wild mice. Splenic CD11c^+^ dendritic cells were proportionately rarer in wild mice compared with laboratory mice (Fig. 5g, Supplementary Table 2).

### Wild mouse NK cells are highly activated

We characterized NKp46^+^ CD3ɛ^−^ NK cells (Fig. 6) as early (stage 1), mid (stage 2), late (stage 3) or fully (stage 4) mature cells by expression of CD27 and CD11b (Fig. 6a). Wild mice had higher proportions of stage 1 and stage 2 cells and lower proportions of stage 3 and stage 4 splenic NK cells, resulting in significantly higher ratios of early/mid-stage NK cells to late/mature NK cells than laboratory mice (Fig. 6b, Table 2). Expression of the recent/early activation marker CD69 was higher on all subsets of wild mouse NK cells compared with laboratory mice (Fig. 6c, Table 2) but—other than on stage 1 cells—expression of the KLRG1 terminal differentiation marker tended to be lower (Fig. 6, Table 2). Together, these data are consistent with activation, self-renewal and homeostatic expansion^16^, and hence higher rates of turnover, of splenic NK cells of wild mice compared with laboratory mice.

We next explored the expression of the Ly49 family of C type lectin regulatory receptors on NK cells (Fig. 6e–h) reasoning that stochastic expression of Ly49 receptor family members on individual NK cells combined with population genetic diversity could lead to NK cell heterogeneity within an individual and extensive variation in NK cell phenotype among individuals^11^. Inhibitory Ly49 receptors recognize self-MHC class I and prevent NK cells from killing healthy cells, whereas Ly49 receptors that recognize pathogen associated ligands lead to NK cell activation and killing of infected cells; the best described example of this is binding of Ly49H to the murine cytomegalovirus (MCMV) m157 glycoprotein which mediates protective immunity to MCMV (ref. 17).

We analysed the expression of two activating receptors (Ly49D and Ly49H) and one inhibitory receptor (Ly49G2). For most C57BL/6 laboratory mice, NK cells expressed Ly49D, Ly49G and Ly49H (Fig. 6e–h) with 5–45% of NK cells expressing each of the receptors, consistent with previous reports^18^. In contrast very few wild mice had any Ly49H^+^ NK cells (10%, *n*=125, ≥1% of Ly49H^+^ cells, Supplementary Data 1), suggesting that the gene encoding this receptor is rare in this wild mouse population or that allelic variation precludes recognition by the anti-Ly49H antibody. We genotyped mice at the *Ly49h* locus for a deletion that is associated with susceptibility to MCMV (ref. 17), finding that 18% of the wild mice were homozygous for this deletion (95% confidence interval 9.5–30%, *n*=98 mice from HW site; frequency of deletion allele 0.42 assuming Hardy–Weinberg equilibrium). This likely partially contributes to the scarcity of Ly49H^+^ NK cells among wild mice, but raises questions about the presence of additional null alleles at the *Ly49h* locus, and whether other receptors can compensate for the lack of Ly49H in wild mice, especially given the high prevalence of MCMV in the wild mouse populations, reported to be 62 and 79% (refs 19, 20). The apparent absence of Ly49H among the wild mice may explain their much more frequent expression of the alternative activating receptor Ly49D and suggests that there may be important differences between wild and laboratory mice in the contributions of NK cells to functional immunity.

We identified three populations of Ly49G2 cells: Ly49G2^−^, Ly49G2^low^ and Ly49G2^high^ (Fig. 6g). Among wild mice most Ly49G2^+^ cells were Ly49G2^low^, whereas in laboratory mice Ly49G2^high^ cells predominated. This suggests the presence of different alleles at the Ly49G2 coding locus in the wild and laboratory populations. Among laboratory mice, interstrain differences in Ly49G receptor expression have been linked to allelic variation in promoter activity^21^ and may influence the threshold for NK cell activation^18^. These data support the idea that there is extensive, hitherto undocumented, allelic diversity among Ly49 receptors with likely important consequences for NK cell function in the wild.

We wanted to understand the balance of expression of activating and inhibitory Ly49 receptors on NK cells, and so compared the proportions of NK cells expressing or not Ly49D and Ly49G2 (Fig. 6e). Wild mice had significantly higher proportions of Ly49D^+^G^−^ cells than laboratory mice whereas laboratory mice had significantly higher proportions of Ly49D^−^G^+^ cells than wild mice (Table 2), suggesting that NK cells of wild mice may have a lower threshold for activation, although this will be heavily influenced by MHC class I genotype and expression of other Ly49 receptors not assayed here. Together these results show that NK cells of wild mice can be, and are, much more readily activated than those of laboratory mice, which may be a necessary response to the high pathogen load of the wild environment.

### Wild mice have reduced cytokine responses to PAMPs

**Figure 7:**
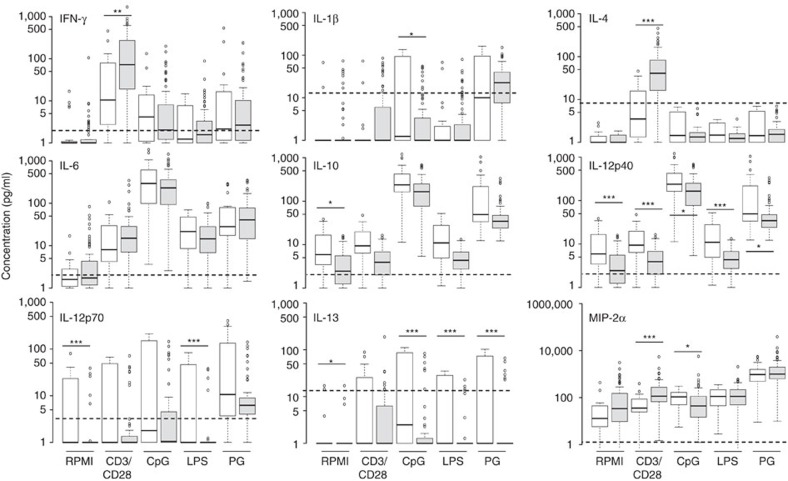
Cytokine production by splenocytes after *in vitro* stimulation. The concentrations of nine cytokines (IFN-γ, IL-1β, IL-4, IL-6, IL-10, IL-12p40, IL-12p70, IL-13, MIP-2α) produced by splenic lymphocytes stimulated with anti-CD3/anti-CD28, CpG, LPS or PG compared with the RPMI control in wild (shaded) and laboratory (unshaded) mice, shown on a log_10_ scale. The box centres are medians, and box limits the 25th and 75th percentiles, the whiskers 1.5 times the interquartile range, and outliers are represented by dots. The dotted horizontal lines show the median lower limit of quantification defined from standard curves across all analysed plates for each cytokine. Asterisks denote significant differences as *P<0.05, **P<0.01, ***P<0.001 (Mann–Whitney *U* test; Supplementary Table 5). Sample sizes are shown in Supplementary Data 1 and Supplementary Table 5.

In light of the highly activated state of the cellular immune system of wild mice we measured the functional immune response by culturing splenocytes in the presence of PAMPs (CpG, the ligand for endosomally expressed TLR9; PG, a TLR2 agonist; bacterial LPS, a ligand for TLR4) and a mitogen (monoclonal antibodies to the T-cell surface molecules CD3 and CD28). Among the 45 comparisons made between wild and laboratory mice (5 culture conditions × 9 cytokines) only 16 significant differences between wild and laboratory mice were observed, and in 13 of these analyte concentrations were significantly lower in the wild mice (Fig. 7, Supplementary Data 1, Supplementary Table 5). Of particular note, wild mice produced significantly less IL-12 (p40 and p70) and less IL-13 than laboratory mice in response to pathogen-related ligands and there was also a trend for IL-10 production to be lower in wild mice, although this was only significant at baseline. These comparatively depressed cytokine responses are in significant contrast to the highly activated cellular immune state of wild mice. We speculate that some form of innate immune tolerance may be operating to limit the degree of inflammation in chronically and highly pathogen-exposed wild mice. The only cytokine responses that were significantly higher in wild mice than in laboratory mice were the IFN-γ, IL-4 and MIP-2α responses to anti-CD3/anti-CD28, which are consistent with the higher proportions of memory and effector T cells in wild mice. These results suggest that innate cytokine responses, and their functional effects, may need to be re-evaluated in laboratory mice.

## Discussion

Laboratory mice are the mainstay of experimental immunology and underpin work that has had a transformative effect on human and animal lives through vaccination and, more recently, immunotherapy. As the human population ages, understanding immune-mediated disease and immunosenescence is of ever increasing importance. For laboratory mice to be useful in understanding these processes, and for treatments and therapies to be effectively translated into human populations, we need to appreciate both the strengths and limitations of the model. Given that the ultimate purpose of the immune system is to provide protection from external environmental threats, the environment in which the immune system is studied is *a priori* likely to have profound effects on its response. The rationale for this work was therefore to understand the immune systems of mice in their natural environment.

This uniquely extensive and detailed immune characterization of wild mice shows that their immune systems are highly activated and antigen-experienced, likely because wild mice are continuously exposed to high levels of environmental antigenic challenge, have high levels of infection and high cumulative exposure to infection over their, often remarkably short, lives. By complete contrast, innate immune function—as measured by cytokine secretion in response to pathogen-associated ligands—is remarkably similar between highly infected wild mice and pathogen-free laboratory mice and, where there are differences, the responses of wild mice are suppressed. We suggest that this suppression of the innate immune response in the wild mice is also driven by their very high levels of antigen exposure, as a homeostatic mechanism to prevent immune-mediated pathology. These observations somewhat go against the emerging concept of ‘trained immunity' or ‘innate immune memory'—that pathogen exposure epigenetically alters innate immune system cells such that they display heightened responses upon a subsequent encounter with the same or similar stimuli^22^. It is also notable that we have identified a novel cell population in wild mice—which we have termed hypergranulocytic myeloid cells—that are apparently absent from laboratory mice. Further work is needed to understand the role of these cells in immune defence and immune regulation. Also of particular interest is our observation of the high frequency of a deletion in the gene coding for the NK cell surface receptor Ly49H. Studies using laboratory mice have shown that deleting this gene results in lethal susceptibility to certain strains of MCMV (ref. 17). However, many naturally occurring strains of MCMV have mutations that abrogate binding to Ly49H, and such mutations can rapidly be selected for by serial passage of MCMV through mice bearing Ly49H (ref. 23) indicating that control of diverse strains of MCMV in the wild may be mediated by a number of different pathways. Our results also suggest that there may be much wider functional diversity in NK cells in wild mice than has hitherto been indicated by studies in laboratory mice.

The status of the immune systems of wild and laboratory mice therefore appears very different. Our data are consistent with the immune systems of wild mice dynamically responding to a large and ever changing diversity of antigens: wild mice seem to be constantly immunologically multi-tasking. In contrast, laboratory mice are likely responding to a very limited antigenic repertoire, allowing greater immunological focus. A typical laboratory mouse experiment will introduce a single pathogen or antigen, often at a high dose and/or with an immune stimulating adjuvant, into an immunologically naive animal. The immune system this antigen encounters is in a largely antigen-inexperienced state, likely acting at baseline cellular turnover rates. In contrast, the same pathogen or antigen in a wild mouse would encounter an immune system that is already highly active and responding to many other antigens. The different states of the immune systems of wild and laboratory mice are therefore predicted to quantitatively and/or qualitatively alter the response to any given antigen. Further, in wild mice, there will be considerable inter-individual differences in these responses. Studies with laboratory mice therefore show what immune responses are possible, but these are not necessarily the immune responses that will actually occur in the real world. The immune response observed in a laboratory mouse is thus just one example of the myriad immune responses that can be generated by outbred, free-living individuals. Considerable caution should therefore be exercised in extrapolating results from laboratory mice to free-living animals and human populations. The research resource we provide here will allow results obtained in laboratory mice to be better contextualized, facilitating the translation of laboratory-based studies to real-world populations.

Laboratory mice are ultimately derived from wild mice, but have been subject to stringent selection during their laboratory history which has, presumably, altered them in many ways. It is likely that selection of laboratory mice for a range of life-history traits (such as rapid growth and reproduction, and docility) has resulted in alteration of a range of traits, including immunological traits. This makes the important point that animals' immune responses need to be considered as one aspect of their wider range of life-history traits, and truly understanding wild animals' immune responses therefore needs to be embedded within the wider context of their life-history biology.

While our work has characterized many immune parameters of wild mice, further characterization (including ‘omics approaches, for example) is warranted to better understand their immune function and its regulation. Although difficult to perform at scale, sequential analyses of individual free-living animals would be highly informative, as would immunization and infection studies in wild mice. Of the commonly used laboratory mouse strains, the C57/BL6 strain used here carries very few mutations that affect immune responses^5^, with the notable exception of *Slc11a1* (formerly *Nramp1*), which confers susceptibility to intracellular bacterial infections; thus, comparison of wild mice with other strains of inbred mice may also be informative. Of relevance to future studies, we found that all of the immunological reagents that we used (which were developed for use in laboratory mice) were suitable for use in wild mice. Although genetic differences between wild and laboratory mice, and among wild mice, may affect the avidity of antibody reagents in no case did we find laboratory reagents that failed to work in wild mice.

While much more remains to be done to fully understand immune function in free living populations, these data provide a sound basis for future studies on wild *Mus musculus domesticus.* Most importantly, the marked among-individual heterogeneity of immune parameters in wild mice provides a rich resource for disentangling associations between genotype, environment and phenotype, and how the immune response contributes to evolutionary fitness.

## Methods

### Mice

This study was approved by the University of Bristol's Animal Welfare and Ethical Review Board. Specified pathogen-free C57BL/6 mice were obtained from Charles River Laboratories (Margate, UK), Harlan (UK) or B&K Universal (UK) and were housed in same-sex groups of five mice per cage, maintained in a 12 h light/12 h dark cycle with environmental enrichment and were fed on commercial rodent diet, *ad libitum* (EURodent diet 22%; PMI Nutrition International, LLC, Brentwood, MO, USA).

Wild mice were live trapped at sites in the southern UK (Fig. 1, Supplementary Table 1) using Longworth traps baited with rolled oats and carrots, left overnight and inspected at least every 24 h (ref. 24). Trapped mice were transferred to a conventional animal house for 2–7 days post capture and maintained under the same conditions as the laboratory reared mice, except that they were housed individually. Mice were trapped between March 2012 and April 2014.

Mice were killed by intraperitoneal injection of sodium pentobarbital. Mice were weighed, measured, and bled out by cardiac puncture and dissected. Blood was collected into a heparinized container, centrifuged at 13,000*g* at 18 °C for 10 min and plasma was aliquoted and stored at −20 °C. Spleens were aseptically removed, weighed and single cell suspensions prepared by homogenization through a 70 μm cell strainer (BD Biosciences); cells were counted by microscopy using a haemocytometer and live cells enumerated by trypan blue exclusion^25^. Eye lens mass was used to determine age according to the formula described elsewhere^26^.

### Infections

We determined the intestinal parasite and ectoparasite fauna of the wild mice as described in ref. 24. We assayed each mouse for evidence of microbial infection using ImmunoComb kits (50MTV103 and 50MIC103, Biogal Galed Labs, Israel), an ELISA-based product to detect antibodies to Corona, Mouse Hepatitis, Sendai, Minute, Noro and Parvo viruses and to *M. pulmonis*. However, because mouse hepatitis virus is a corona virus and the manufacturer's data sheet lacked clarity as to the antigens used in the assay, corona virus data were excluded to avoid over estimation of the number of viruses present in any particular sample.

### Genotyping

Genomic DNA was extracted from 5 mm tail tips which were digested with 0.8 mg ml^−1^ proteinase K (Life Technologies) in a digestion buffer (30 mM Tris pH 8, 10 mM EDTA pH 8, 1% w/v SDS pH 8) for 12 h at 56 °C. The resulting solution was treated with 0.4 mg ml^−1^ RNAseA (Life Technologies), DNA was purified using standard phenol/chloroform extraction, precipitated in ethanol, resuspended in TE buffer (1 mM EDTA pH 8, 10 mM Tris pH 8) and quantified by Qubit (Life Technologies).

A total of 167 wild mice from site HW (Fig. 1, Supplementary Data 3) and two C57BL/6 (designated L88 and L90) mice were genotyped at 1,168 autosomal loci using the GoldenGate Mouse MD mouse chip (GT-18-131, Illumina) following the manufacturer's instructions. The loci on this chip were chosen to differentiate C57BL/6 mice from other commonly used mouse strains. Technical replicates of the laboratory mouse DNA (15 replicates per sample) indicated that the genotyping error rate across the 2,336 nucleotides was <1%. Our genotyping of C57BL/6 mice showed perfect concordance with published data ( http://support.illumina.com/array/array_kits/mouse_md_linkage/downloads.html) for this strain.

We constructed a pairwise distance matrix of the number of genotyped nucleotide differences among individuals, and included reference genotypes for other laboratory strains (C57BL/6J, NOD/Ltj, 129S1/SvlmJ, CBA/J, AKR/J, FVB/NJ, C3H/HeJ, BALB/cJ, DBA/2J, SJL/J) provided by Illumina ( http://support.illumina.com/array/array_kits/mouse_md_linkage/downloads.html). The distance matrix was then used to construct a nearest neighbour joining tree in MEGA6 (ref. 27).

### Serum protein ELISA

Serum concentrations of haptoglobin, SAP and AAT were determined using in-house sandwich ELISAs. Briefly, 96-well microtitre plates were coated with 50 μl per well of goat polyclonal anti-mouse haptoglobin (Immunology Consultants Laboratory, Portland, USA; 1 in 4,000), rabbit polyclonal anti-SAP (Calbiochem, Watford, UK; 1 in 6,000) or chicken polyclonal anti-AAT (Immunology Consultants Laboratory, Portland, USA; 1 in 6,000) diluted in PBS coating buffer and incubated overnight at 4 °C. Plates were washed twice with PBS-Tween (0.05% v/v Tween-20 in PBS) washing buffer and blocked for 2 h at room temperature with 1% w/v bovine serum albumin in PBS-Tween. The plates were washed twice, 50 μl of each test serum (diluted 1 in 10,000–1,00,000 for haptoglobin; 1 in 400 for SAP; 1 in 80,000 for AAT) in blocking buffer added to duplicate wells, incubated overnight at 4 °C and washed. For analyte detection, 50 μl of affinity purified goat polyclonal antibody to SAP, haptoglobin or AAT (Immunology Consultants Laboratory, Portland, USA; biotinylated in-house using NHS-LC-Biotin, Cayman Chemical Company Co, Ann Arbor, USA), diluted to 0.1–0.5 μg ml^−1^ in PBS-Tween, added to each well and incubated for 1 h at room temperature. Plates were washed, incubated with 50 μl per well of streptavidin-horseradish peroxidase (0.125 μg ml^−1^; Invitrogen, Paisley, UK) in PBS-Tween for 30 min at room temperature, washed again and developed with 100 μl per well of tetramethylbenzidine solution (Tebu-bio, Peterborough, UK) for 15–20 min in the dark at room temperature. The reaction was stopped by the addition of 25 μl per well of 2 M H_2_SO_4_ and the plates were read at 450 nm on a Dynex MRXII plate reader (Dynex Technologies, Worthing, UK).

SAP, haptoglobin and AAT concentrations were determined by interpolation of optical density units for standard curves generated from purified proteins or standards. SAP was purified from normal mouse serum using phosphorylethanolamine-sepharose 4B affinity chromatography and anion exchange (DE-52) chromatography, as previously described^28^. Standards for haptoglobin and AAT were purchased from Immunology Consultants Laboratory.

Serum IgG and IgE ELISAs were performed using Ready-SET-Go Kits (88-50400 and 88-50460) from eBioscience (Hartfield, UK), following the manufacturer's instructions with the exception that we diluted the test samples for the wild mice 1 in 400,000 for IgG and 1 in 200 for IgE respectively^10^.

### Faecal IgA ELISA

Faeces were removed from the hindgut of mice, weighed and dissolved in 50 μl PBS supplemented with a protease inhibitor cocktail at a final concentration of 20% v/v (SIGMAFASTTM Protease Inhibitor cocktail Tablets, EDTA-free) to form a slurry that was left at room temperature for 1 h. The samples were then centrifuged at 13,000*g* for 10 min at 4 °C, and the supernatant removed. The IgA concentration of these supernatants was measured using a commercially available mouse IgA ELISA kit following the manufacturer's instructions (Mouse IgA ELISA Ready-SET-Go, 88-50450, eBioscience). Faecal supernatant samples were serially diluted starting from a 1:500 dilution (in PBS with 1% v/v Tween and 10% w/v BSA). Standard curves were constructed using the mouse IgA supplied with the kit, and this was used to interpolate the IgA concentrations in our samples expressed as the μg concentration per g of faeces.

### Spleen cell cultures

Spleen cells were suspended in complete culture medium (RPMI1640 supplemented with 10% v/v FCS, 100 U ml^−1^ penicillin/streptomycin and 2 mM L-glutamine; all Life Technologies, Paisley, UK) at a concentration of 1.5 × 10^6^ cells per ml and 0.1 ml aliquots were dispensed into each well of 96-well round-bottomed tissue culture plate (Greiner Bio-One, Stonehouse, UK). One of the following stimulants (0.1 ml) was added to each well: anti-CD3/anti-CD28 (each at 5 μg ml^−1^; eBioscience, Hartfield, UK), CpgG1826 (at 1 μg ml^−1^; MWG Eurofins, Ebersberg, Germany), PG (at 10 μg ml^−1^; Sigma, Poole, UK), LPS (2.5 μg ml^−1^); 0.1 ml complete culture medium was used as a negative control. Cultures were incubated at 37 °C in 5% CO_2_ for 15 h, centrifuged at 400*g* for 5 min and approximately 0.15 ml of supernatant was removed, divided into two aliquots and stored at −80 °C until assayed for cytokines by bead array.

### Flow cytometry

Freshly isolated spleen cells were stained for leucocyte surface markers with intracellular staining for Foxp3 following permeabilization of cells^25^. Samples were analysed on an LSR-II flow cytometer (BD Biosciences) with FlowJo software (Treestar, Ashland, USA). Details of the antibodies and their combination into staining panels are provided in Supplementary Table 6. Gating strategies for lymphocyte subpopulations are shown in Supplementary Fig. 1.

### *Ly49h* genotyping

To complement the flow cytometry analysis of Ly49 markers, we genotyped mice at the *Ly49h* locus (also known as *Klra8* (ref. 29)), where a deletion has previously been associated with inducing susceptibility to mouse cytomegalovirus infection^18^. We genotyped 98 randomly selected HW mice at the *Ly49h* locus by PCR as described in ref. 30 using primer pair D6Ott174 that lies within the putative deletion (such that non-amplification indicates the mouse is homozygous for the deletion) combined with amplification using the primer pair D6Ott175 as a control for amplification of this locus. We used DNA from control mice, specifically C57BL/6 and B65.BXD8 without and with the deletion^30^, respectively, as controls.

### Cytokine bead arrays

Cytokine concentrations in culture supernatants were assayed using Bioplex Pro kits (M60-009RDPD & MD0-00000EL, Bio-Rad, UK) following the manufacturer's instructions, and analysed on a Luminex 100 (LuminexCorp, Austin, USA) running Bioplex Manager software. Initially supernatants from 31 wild and 10 laboratory mice were analysed for 32 cytokines and chemokines (Supplementary Data 4) and these data used to identify analytes where concentrations above the assay's limit of detection were observed in at least some animals and where the data were not highly correlated with other analytes. This resulted in the selection of nine highly informative analytes comprising IL-1β, IL-4, IL-6, IL-10, IL-12p40, IL-12p70, IL-13, IFN-γ and MIP-2α (Fig. 7). Data that were below the assay's range were set to values of 0.001 for the purposes of statistical analyses.

### Statistical analyses

Owing to technical or sample volume limitations, not all parameters could be measured for all mice and thus sample sizes vary on a test-by-test basis and are reported either alongside the data or in the degrees of freedom. Data that were normally distributed (body mass; spleen mass:body mass ratio) or that could be normalized (age; log_10_ transformed) were analysed using general linear models. All other data were analysed using repeated non-parametric tests (Mann–Whitney *U* tests, unless otherwise indicated). The comparison of wild and laboratory mice compares 181 wild mice from site HW and 64 laboratory mice (LMC 1–4, 9–41, 64, 65, 68–82, 85–93, 95) (Supplementary Data 1).

Owing to the different sample sizes, and the differing proportions of male and female mice between the laboratory and wild animal groups, separate, univariate general linear models were used to analyse the effect of sex on the age of wild mice and also the effect of animal group (that is, wild versus laboratory) and the effect of sex within these groups on body mass and spleen mass:body mass ratio. Mann–Whitney *U* tests were used to compare spleen mass and viable spleen cell number between animal group and sex within group.

The remaining variables were tested for any effect of sex within each animal group. Where variables differed significantly by sex for either group, we divided the mice into four groups: laboratory female, laboratory male, wild female and wild male. Kruskal–Wallis H tests were then applied with *post hoc* pairwise comparisons (Dunn–Bonferroni), controlling for Type I error inflation. Associations between immunological and morphometric parameters among wild mice were assessed by Pearson correlations (two-tailed) on untransformed data. Finally, Mann–Whitney *U* tests were used to compare differences in cytokine production between groups. All analyses were conducted in SPSS v.21 (IBM Corp., New York, USA). Box plots were produced using R software via the website, boxplot.tyerslab.com.

### Data availability

Genotype data referenced in this study are available in Mouse MD Linkage Panel Support^31^. The data that support the findings of this study are available within the paper and its Supplementary Information Files.

## Additional information

**How to cite this article:** Abolins, S. *et al*. The comparative immunology of wild and laboratory mice, *Mus musculus domesticus*. *Nat. Commun.* **8,** 14811 doi: 10.1038/ncomms14811 (2017).

**Publisher's note:** Springer Nature remains neutral with regard to jurisdictional claims in published maps and institutional affiliations.

## Supplementary Material

Supplementary Information Supplementary Figure and Supplementary Tables

Supplementary Data 1 Complete data set of immune measures of wild and laboratory mice. (a) immunoglobulins, serum proteins and morphometrics (b1-3) splenocyte populations analysed by flow cytometry and (c) cytokine responses of *in vitro* stimulated splenocytes. In all column A is the mouse ID shown as Wild Mouse (WM) or Lab Mouse Control (LMC). In (a) Column B shows the site from where the wild mouse was caught (Supplementary Table 1) or the laboratory mouse strain. All laboratory mouse strains were obtained in Bristol except those labelled as LSHTM which were from the London School of Hygiene and Tropical Medicine. LMC_5-8 are C57BL/6 with an unrecorded, non-immunological genetic manipulation. Column D shows females' pregnancy status where 1 is pregnant and 2 is not pregnant; column E is animals' mass when trapped; column F their age in weeks; column G spleen mass in g, column H the number of viable spleen cells; columns I - N the immunoglobulin and serum protein concentrations that are in concentrations of μg/mL (μg/g for faecal IgA); SAP is serum amyloid P; AAT is alpha-1 antitrypsin. For (b1) shows median fluorescence index (MFI) of the relevant markers; (b2) the proportionate presence of cellular sub-populations, and row 2 the parent cellular populations from which the other proportionate cellular populations (row 1) were calculated; (b3) the number of cells of different cellular populations. In (b1)-(b3) the row labelled 'Panel' shows the relevant FACS panel as Supplementary Table 6. Where there are an absence of data the reasons are shown as ND - no FACS analysis performed; NC - marker combination not present in this panel / staining; NS – not stained; TFC – too few cells for reliable sub-population analysis; OUT — outlier; NA — other reason. In (c) the cytokine analysed and the stimulation are shown in rows 1 and 2, respectively. Note for the PIC stimulation not all samples were stimulated. Data that were out of range (OOR) are shown as below (<) or above (>) the range; data indicted by * are where the value is extrapolated beyond the limits of the standard curves. OOR< data were set to values of 0.001 for the purposes of quantitative analyses. All concentrations are in pg/mL.

Supplementary Data 2 Correlation matrix of wild mouse morphometrics, infection status and immune measures. For each measure the Pearson correlation (two-tailed) value *r*, the *P* value and the relevant sample size are shown. Cells are colour coded such that > 0.01 *P* < 0.05 is green and *P* < 0.01 is red. The results for male mice are above the black diagonal, female mice below. The column and row headings are as for Table 1, Table 2 and Supplementary Table 2 for morphometric and immune measures. Mites and Nematodes refers to the intensity of infection of mice; Virus refers to the number of virus infections, both as Supplementary Table 3. Results are for the HW sample site.

Supplementary Data 3 Wild mouse multilocus genotypes. (a) shows the 1168 locus genotypes of mice where column A is the locus name, column B the chromosome the locus is on, column C the position of the locus on the chromosome. Row 1 shows the source of the mice either as wild mice from HW or laboratory mouse strains shown as L, and row 2 is the individual number of the wild mouse (prefix WM). Two individual C57BL/6 laboratory mouse controls (L88 and L90) were each genotyped 15 times with the repeat number shown by the third and four digits of their row 2 designation. Genotype data provided by Illumina for ten other laboratory mouse strains are also shown. (b) shows the pair-wise distance matrix for HW wild mice where columns A and B shows individual pairs of mice including laboratory mouse controls (as in (a)) and column C the distance between them shown as the number of nucleotide differences of a maximum of 2336 nucleotides.

Supplementary Data 4 Preliminary characterisation of cytokines and chemokines. For wild and laboratory mice where column A is the mouse ID shown as Wild Mouse (WM) or Lab Mouse Control (LMC), Column B shows the site from where the wild mouse was caught (Supplementary Table 1), or that mice were laboratory mice. Column D shows what was used to stimulate the cells as anti-CD23/anti-CD28 (CD3CD28), Cpg1826 (CPG), Flagellin (FLAG), Lipopolysaccharide (LPS), Peptidoglycan (PG), Poly(I:C) (PIC) or RPMI culture medium (RPMI). The cytokine analysed is shown in row 1. Data that were out of range (OOR) are shown as below (<) or above (>) the range; data indicted by * are where the value is extrapolated beyond the limits of the standard curves. OOR< data were set to values of 0.001 for the purposes of quantitative analyses. All concentrations are in pg/mL. Eotaxin — Eosinophil chemotactic protein, also known as CCL11 chemokine (C-C motif) ligand 11; GCSF — Granulocyte colony stimulating factor; GMCSF — Granulocyte macrophage colony stimulating factor; KC — Keratinocyte chemoattractant, also known as CXCL1 chemokine (C-X-C motif) ligand 1; MCP-1 — Monocyte chemoattractive protein-1, also known as CCL2 chemokine (C-C motif) ligand 2; MIP-1a — Macrophage inflammatory protein-1 α, also known as CCL3 chemokine (C-C motif) ligand 3; MIP-1β — Macrophage inflammatory protein — beta, also known as CCL4 chemokine (C-C motif) ligand 4; RANTES — Regulated on activation, normal T expressed and secreted, also known as CCL5 chemokine (C-C motif) ligand 5; Basic FGF — Basic fibroblast growth factor, also known as FGF2; LIF — Leukaemia inhibitory factor (IL-6 family cytokine); MCSF — Macrophage colony stimulating factor, also known as CSF-1 colony stimulating factor 1; MIG — Monokine induced by interferon gamma, also known as CXCL9 chemokine (C-X-C motif) ligand 9; MIP-2α — Macrophage inflammatory protein-2 α, also known as CXCL2 chemokine (C-X-C motif) ligand 2; PDGF-BB — Platelet derived growth factor-BB; VEGF — Vascular endothelial growth factor.

## Figures and Tables

**Table 1:** The body characteristics and serum protein concentrations of wild mice and their comparison to laboratory mice.

**Parameter**	**Laboratory**			**Wild**			
	**Female**	**Male**	**Female versus Male**	**Female**	**Male**	**Female versus Male**	**Lab. versus** **Wild**
Age (weeks)				7.2 (1.4–39.5; 80)	6.2 (1.0–20.9; 99)	*F*_1, 178_=2.426	
Body length (mm)				67 (50–80; 81)	70 (48–86; 100)	*F*_1, 180_=3.448	
Body mass (g)	20.5 (14.1–40.4; 37)	25.75 (19–32; 24)	*F*_1, 60_=8.35**	12.9 (3.3–24.9; 81)	13.9 (4.7–20.4; 100)	*F*_1, 180_=0.204	*F*_1, 241_=193.2***
Spleen mass (mg)	82 (48–132; 38)	76 (53–112; 24)	*U*=408	25 (2–200; 78)	24 (4–644; 99)	*U*=3891	*U*=10575***
Viable spleen cells (millions)	101 (0.43–299; 38)	119 (2.66–204; 24)	*U*=575	20.5 (1.40–78.20; 64)	20.6 (4.6–86.2; 83)	*U*=2718	*U*=8575***
Spleen mass: body mass ratio	0.0039 (0.0025–0.0053; 36)	0.0031 (0.0022–0.0041; 24)	*F*_1, 59_=22.74***	0.0020 (0.0030–0.0044; 78)	0.0020 (0.0004–0.0081; 97)	*F*_1, 174_=0.005	*F*_1, 234_=116.67***
Serum IgG (μg ml^−1^)	358 (9–13326; 39)	282 (47–1912; 24)	*U*=376	6889 (221–35784; 79)	5716 (200–35642; 96)	*U*=3374	*U*=517***
Serum IgE (μg ml^−1^)	0.42 (0.01–15.21; 34)	0.33 (0.03–0.69; 17)	*U*=193	132 (8–753; 79)	81 (1–832; 96)	*U*=2685***	*H*=123.1***, LF<WF***, LF<WM***, LM<WF***, LM<WM***
Faecal IgA (μg g^−1^)	248 (170–379; 7)	163 (130–283; 5)	*U*=7	199 (5–1168; 45)	268 (2–1178; 64)	*U*=1534	*U*=621
SAP (μg ml^−1^)	1.29 (0.49–2.58; 33)	0.77 (0.30–1.96; 17)	*U*=101***	2.06 (0.11–6.43; 78)	1.68 (0.14–5.43; 96)	*U*=3225	*H*=19.8***, LM<WF***, LM<WM**
Haptoglobin (μg ml^−1^)	0.505 (0.15–315; 40)	0.54 (0.54–424; 23)	*U*=749***	4.87 (0.36–1190; 78)	5.34 (0.48–571; 96)	*U*=3630	*H*=56.5***, LF<WF***, LF<WM***
AAT (μg ml^−1^)	248 (4–488; 37)	286 (26–456; 19)	*U*=436	272 (2–596; 77)	263 (3–748; 95)	*U*=3631	*U*=4621

The table shows the body characteristics and serum protein concentrations for wild and laboratory mice, shown as medians (range; *n*). Statistical results show comparisons between sex within animal source (that is, laboratory or wild mice), and between animal source. Where there exists a significant difference between sex, mice were grouped by sex and animal source as: laboratory female, laboratory male, wild female and wild male (LF, LM, WF, WM, respectively), with Kruskal–Wallis *H*-tests (and *post hoc*, Dunn–Bonferonni, pairwise comparisons) conducted with significant differences reported. Where data were normal (that is, body length, body mass and spleen mass: body mass ratio) or could be transformed to normal (log_10_ transformation of age; untransformed data shown), the results of univariate GLMs are reported. All non-normal data were analysed by Mann–Whitney *U*-tests, unless stated otherwise. Asterisks denote significant differences as *P<0.05, **P<0.01, ***P<0.001.

**Table 2:** Characterization of natural killer cell populations of wild mice and their comparison to laboratory mice.

**Parameter**	**Laboratory**			**Wild**			
	**Female**	**Male**	**Female versus Male**	**Female**	**Male**	**Female versus Male**	**Lab. versus Wild**
^1^NKp46^+^ (%)	1.9 (0.5–4.7; 36)	3.1 (0.2–7.4; 24)	*U*=636**	1.5 (0.7–3.5; 60)	1.5 (0.2–5.4; 77)	*U*=2419	*H*=28.3***, LF>WF*, LM>WF***, LM>WM***
^2^NKp46^+^ stage 1 (%)	9.2 (3.2–38.0; 36)	6.3 (2.5–27.2; 22)	*U*=147***	10.4 (4.6–39.1; 53)	12.3 (3.6–45.4; 64)	*U*=1575	*H*=22.4***, LM<WF***, LM<WM***
^2^NKp46^+^ stage 2 (%)	17.9 (8.3–88.4; 36)	18.3 (10.4–93.3; 22)	*U*=508	29.6 (8.0–71.0; 53)	36.2 (15.2–71.0; 64)	*U*=2031	*U*=1362***
^2^NKp46^+^ stage 3 (%)	29.7 (0.06–55.8; 36)	34.7 (0.06–50.8; 22)	*U*=431	21.7 (7.3–50.0; 53)	20.3 (5.4–45.5; 64)	*U*=1747	*U*=4656***
^2^NKp46^+^ stage 4 (%)	38.4 (0.08–58.7; 36)	34.2 (0.06–52.9; 21)	*U*=298	32.4 (4.3–58.9; 53)	26.6 (5.1–54.1; 64)	*U*=1423	*U*=4431**
NK stages 1&2 : 3&4 ratio	0.40 (0.13–665; 36)	0.34 (0.16–1666; 22)	*U*=363	0.80 (0.26–7.33; 53)	0.99 (0.25–6.33; 64)	*U*=1954	*U*=1542***
NKp46^+^ stage 1 CD69 (MFI)	94 (35–335; 36)	92 (51–207; 21)	*U*=352	132 (38–282; 53)	150 (81–525; 64)	*U*=2166**	*U*=1119***
NKp46^+^ stage 2 CD69 (MFI)	232 (71–533; 36)	126 (76–254; 21)	*U*=214**	203 (50–681; 53)	202 (110–991; 64)	*U*=1884	*H=*14.8**, LM<WF*, LM<WM***
NKp46^+^ stage 3 CD69 (MFI)	119 (57–515; 36)	85 (69–1104; 21)	*U*=316	221 (50–916; 53)	261 (120–1142; 64)	*U*=2105*	*H*=54.2***, LF<WF***, LF<WM***, LM<WF***, LM<WM***
NKp46^+^ stage 4 CD69 (MFI)	85 (30–207; 36)	65 (45–120; 20)	*U*=234*	200 (67–750; 53)	212 (89–984; 64)	*U*=2082*	*H*=89.0***, LF<WF***, LF<WM***, LM<WF***, LM<WM***
NKp46^+^ stage 1 KLRG1 (MFI)	91 (67–324; 18)	83 (47–200; 19)	*U*=135	92 (19–261; 43)	106 (17–341; 46)	*U*=1113	*U*=1574
NKp46^+^ stage 2 KLRG1 (MFI)	30 (1–44; 15)	20 (7–72; 17)	*U*=136	9 (1–38; 24)	9 (1–65; 27)	*U*=350	*U*=1256***
NKp46^+^ stage 3 KLRG1 (MFI)	133 (79–351; 18)	93 (57–967; 19)	*U*=110	57 (5–280; 41)	71 (10–1585; 42)	*U*=105	*U*=2334***
NKp46^+^ stage 4 KLRG1 (MFI)	1358 (727–1978; 18)	1121 (602–2730; 19)	*U*=114	926 (55–2872; 43)	1143 (99–3895; 49)	*U*=1279	*U*=2158**
^2^NKp46^+^ Ly49D^+^ (%)	48.5 (0.6–81.0; 31)	54.6 (47.2–60.5; 18)	*U*=421**	71.1 (41.8–80.4; 56)	71.3 (52.8–86.7; 69)	*U*=2039	*H*=80.9***, LF<WF***, LF<WM***, LM<WF***, LM<WM***
^2^NKp46^+^ Ly49G2^+^ (%)	39.6 (0.2–96.3; 31)	47.6 (44.2–53.1; 18)	*U*=424**	30.3 (12.9–59.4; 56)	29.4 (6.7–66.3; 69)	*U*=1810	*H*=34.2***, LF>WM*, LM>WF***, LM>WM***
^2^NKp46^+^ Ly49H^+^ (%)	46.0 (0.1–88.9; 31)	58.3 (44.9–64.8; 18)	*U*=475***	0.13 (0.0–16.7; 56)	0.12 (0.0–46.0; 69)	*U*=1774	*H*=97.8, *P<*0.001***, LF>WF***, LF>WM***, LM>WF**, LM>WM***
^2^NKp46^+^ Ly49D^+^G2^-^ (%)	23.55 (0.55–49.50; 31)	24.41 (18.45–27.99; 18)	*U*=343	44.57 (15.62–63.68; 56)	46.00 (20.95–68.29; 69)	*U*=2022	*U*=431***
^2^NKp46^+^ Ly49D^+^G2^+^ (%)	23.30 (0.07–96.30; 31)	31.35 (26.70–33.82; 18)	*U*=424**	26.76 (11.60–53.10; 56)	25.40 (5.79–61.72; 69)	*U*=1793	*H*=11.9**, LM>WM*
^2^NKp46^+^ Ly49D^-^G2^+^ (%)	15.34 (0.00–38.65; 31)	16.70 (13.27–22.35; 18	*U*=372	4.18 (0.74–6.80; 56)	3.88 (0.31–7.62; 69)	*U*=1722	*U*=5765***
^2^NKp46^+^ Ly49D^-^G2^-^ (%)	35.09 (0.02–75.40; 31)	26.76 (23.97–32.80; 18)	*U*=111***	23.24 (14.68–56.24; 56)	24.03 (11.03–41.76; 69)	*U*=1981	*H*=37.9***, LF>WF***, LF>WM***

The table shows the natural killer cell populations for wild and laboratory mice, shown as medians (range; *n*). Statistical results show comparisons between sex within animal source (that is, laboratory or wild mice), and between animal source. Where there exists a significant difference between sex, mice were grouped by sex and animal source as: laboratory female, laboratory male, wild female and wild male (LF, LM, WF, WM, respectively), with Kruskal–Wallis *H*-tests (and *post hoc*, Dunn–Bonferonni, pairwise comparisons) conducted with significant differences reported. All non-normal data were analysed by Mann–Whitney *U*-tests, unless stated otherwise. Asterisks denote significant differences as *P<0.05, **P<0.01, ***P<0.001. Superscripts define cell populations as a percentage of: ^1^spleen cells; ^2^NK cells.
