# Supplementary material for: The comparative immunology of wild and laboratory mice, Mus musculus domesticus
Source: Nat Commun. 2017 May 3;8:14811. doi: 10.1038/ncomms14811 (PMC5418598; doi:10.1038/ncomms14811)
Supplement: Supplementary Information — Supplementary Figure and Supplementary Tables [file ncomms14811-s1.pdf]

(a) Phenotyping of lymphocytes

(b) Phenotyping of dendritic cells and other myeloid cells

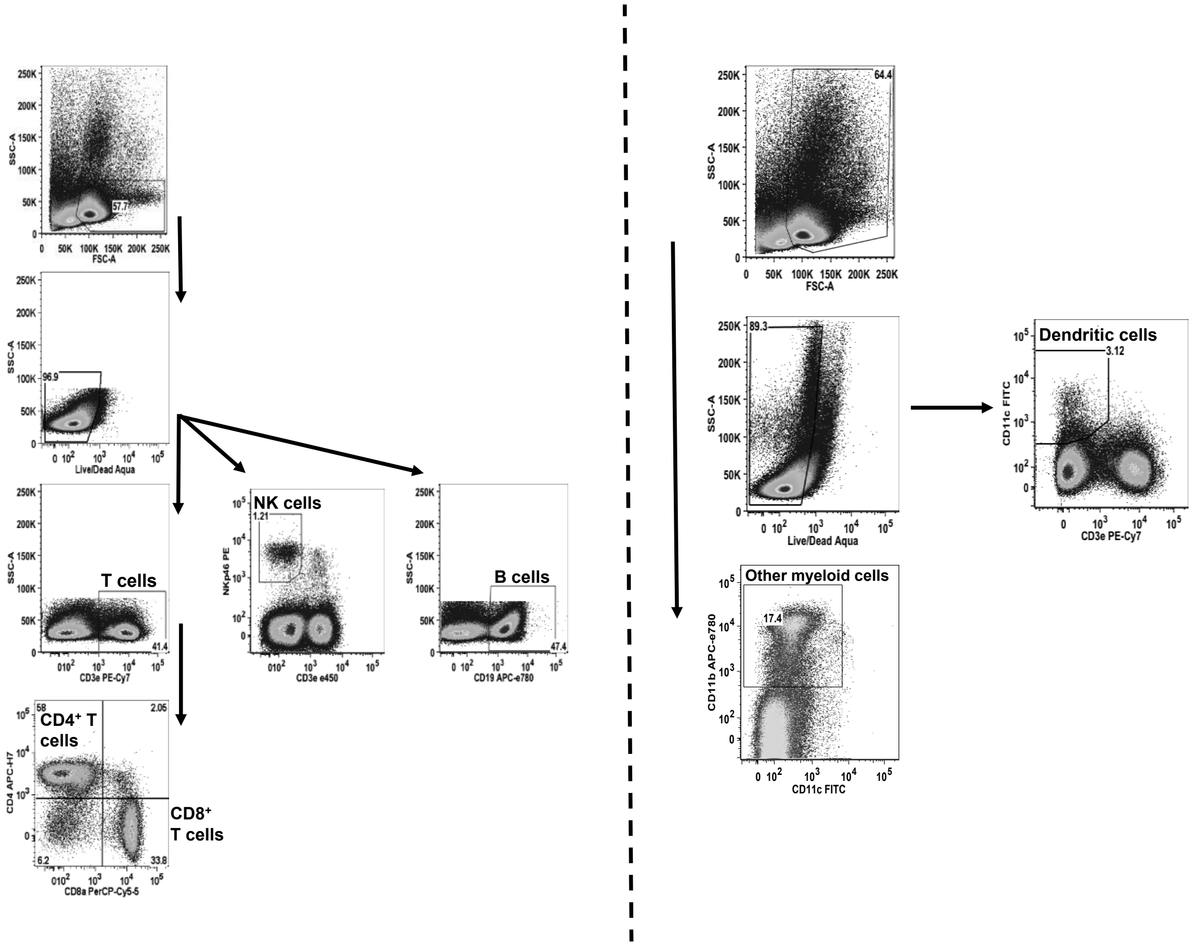

Supplementary Figure 1. Flow cytometry gating strategies. (a) For lymphocytes, live cells are gated from the lymphocyte population (FSC *vs.* SSC plot); T cells are CD3ε $^{+}$ cells, which are further subdivided into CD4^{+} or CD8^{+}. NK cells are NKp46^{+} CD3ε^{-} and B cells are CD19^{+}. (b) For myeloid cells, dendritic cells are CD11c^{+} CD3ε $^{-}$ and other myeloid cells are CD11b^{+} CD11c^{-}.

Site code	OS grid reference	Site description	Number of mice
HW	ST 506 671	Mixed arable and beef farm	181
PH	ST 532 595	Dairy farm	66
JB	ST 595 652	Stables and livery	36
BM	ST 523 686	Grain merchant and mill	35
GL	SO 786 087	Beef farm	33
SK	SM 737 050	Island, bird observatory	33
WF	SO 882 004	Dairy farm and cheese producer	18
LU	TQ 290 812	London Underground[1]	18
ST	ST 524 688	Beef and dairy farm	15
PF	ST 554 710	Dairy farm	10
WT	SO 818 058	Dairy farm	8
SP	ST 665 670	Dairy farm	7

Supplementary Table 1. Wild mouse sample sites. OS is Ordnance Survey, UK.

[1]WM_21, 22 Central Line, Oxford Circus station; WM_261–274 and 441, Central Line, Holborn station; WM_442 Piccadilly Line, Leicester Square station.

Parameter	Laboratory			Wild			
	**Female**	**Male**	**Female *vs.* Male**	**Female**	**Male**	**Female *vs.* Male**	**Lab. *vs.* Wild**
**[1]CD3$^+$ (%)**	29.5 (21.5 - 66.2; 39)	35.9 (25.0 - 57.8; 24)	*U* = 619 *	45.9 (30.5 - 70.0; 53)	45.7 (17.6 - 82.0; 71)	*U* = 1535	*H* = 49.8 ***, LF < WF***, LF < WM***, LM < WF***, LM < WM***
**[2]CD4$^+$ (%)**	55.7 (43.9 - 70.5; 39)	55.6 (48.1 - 64.8; 24)	*U* = 505	61.4 (43.7 - 80.4; 53)	59.7 (36.6 - 80.2; 71)	*U* = 1520	*U* = 1882 ***
**[3]Naive CD4$^+$ (%)**	62.0 (20.6 - 80.5; 39)	70.2 (14.5 - 80.9; 24)	*U* = 565	41.1 (6.2 - 73.8; 53)	44.2 (5.6 - 68.8; 71)	*U* = 2064	*U* = 6059 ***
**[3]Memory CD4$^+$ (%)**	4.4 (1.4 - 17.1; 39)	4.5 (1.2 - 14.8; 24)	*U* = 462	6.3 (1.4 - 17.1; 53)	6.1 (0.4 - 51.0; 71)	*U* = 1751	*U* = 2789 ***
**[3]Effector CD4$^+$ (%)**	11.5 (4.3 - 36.9; 39)	12.7 (6.4 - 34.4; 24)	*U* = 469	33.6 (9.4 - 62.4; 53)	30.1 (2.7 - 54.0; 71)	*U* = 1724	*U* = 817 ***
**[2]CD8$^+$ (%)**	38.3 (23.2 - 48.7; 39)	37.5 (30.3 - 43.7; 24)	*U* = 423	30.4 (13.7 - 49.0; 52)	32.4 (11.3 - 53.8; 71)	*U* = 1787	*U* = 5831 ***
**[4]Naive CD8$^+$ (%)**	64.5 (24.0 - 78.0; 37)	68.7 (42.6 - 81.1; 24)	*U* = 573	52.1 (11.6 - 70.8; 52)	52.6 (4.8 - 76.6; 71)	*U* = 1900	*U* = 5671 ***
**[4]Memory CD8$^+$ (%)**	19.6 (5.9 - 36.5; 37)	19.4 (3.9 - 29.4; 24)	*U* = 439	16.3 (5.7 - 34.6; 52)	13.3 (1.3 - 29.0; 71)	*U* = 1447 *	*H* = 13.3 **, LF > WM**, LM > WM*
**[4]Effector CD8$^+$ (%)**	5.9 (2.0 - 16.5; 37)	4.4 (2.4 - 20.7; 24)	*U* = 337	19.7 (3.0 - 49.2; 52)	19.0 (3.6 - 48.0; 71)	*U* = 2027	*U* = 611***
**Terminally differentiated (KLRG1$^+$) CD8$^+$ (%)**	1.18 (0.01 – 14.0; 37)	1.3 (0.15 - 9.8; 24)	*U* = 517	2.2 (0.00 - 20.5; 55)	3.0 (0.00 - 35.0; 68)	*U* = 2229	*U* = 2728**
**CD4$^+$: CD8$^+$ ratio**	1.47 (0.93 - 3.04; 39)	1.44 (1.20 - 2.14; 24)	*U* = 486	2.1 (1.12 - 5.43; 50)	1.89 (0.98 - 7.07; 66)	*U* = 1515	*U* = 1535***
**[3]CD25$^+$ Foxp3$^+$ (Treg) (%)**	5.2 (1.8 - 11.2; 36)	4.4 (0.1 - 9.4; 23)	*U* = 323	5.4 (0.7 - 11.9; 52)	6.4 (1.1 - 13.3; 63)	*U* = 1649	*U* = 2623*
**Effector CD4$^+$: Treg ratio**	2.69 (1.16 - 6.69; 36)	2.76 (1.79 - 206.27; 23)	*U* = 512	7.08 (1.94 - 50.54; 49)	4.86 (2.02 - 29.68; 62)	*U* = 1220	*U* = 1179***
**Effector CD8$^+$: Treg ratio**	0.96 (0.30 - 24.90; 33)	1.25 (0.41 - 46.70; 19)	*U* = 359	3.78 (0.50 - 38.47; 49)	3.12 (0.57 - 30.38; 62)	*U* = 1308	*U* = 947***
**Terminally**	0.24 (0.00 - 2.17; 35)	0.43 (0.08 - 60.23; 23)	*U* = 529 *	0.55 (0.00 - 7.73; 49)	0.74 (0.00 - 22.15; 61)	*U* = 1670	*H* = 9.7 *, LF < WM*

**differentiated CD8$^+$: Treg ratio**							
**[1]CD19$^+$ (%)**	46.9 (15.8 - 59.7; 37)	48.8 (18.6 - 61.6; 23)	*U* = 456	43.9 (24.6 - 62.6; 58)	42.6 (18.9 - 60.0; 78)	*U* = 2147	*U* = 4676
**[5]Naive CD19$^+$ (%)**	73.9 (48.6 - 90.7; 37)	81.9 (53.6 - 86.6; 23)	*U* = 516	82.2 (65.1 - 89.7; 57)	82.6 (65.4 - 92.1; 76)	*U* = 2356	*U* = 2573 ***
**[5]Memory CD19$^+$ (%)**	15.26 (6.7 - 27.4; 37)	13.0 8.4 - 28.5; 23	*U* = 340	11.1 (6.2 - 21.9; 57)	11.9 (5.5 - 18.7; 75)	*U* = 2213	*U* = 5180 ***
**[5]Germinal centre CD19$^+$ (%)**	0.4 (0.01 - 1.20; 37)	0.2 (0.05 - 1.29; 23)	*U* = 282 *	0.6 (0.14 - 6.93; 57)	0.7 (0.09 - 6.9; 75)	*U* = 2314	*H* = 32.6 ***, LF < WM**, LM < WF***, LM < WM***
**CD19$^+$ PNA (MFI)**	990 (202 - 3161; 36)	1719 (447 - 2909; 23)	*U* = 522	2123 (172 - 4297; 59)	1933 (232 - 5330; 74)	*U* = 19001	*U* = 2670 ***
**CD19$^+$ MHC II (MFI)**	29209 (199 - 40838; 37)	29200 (380 - 37683; 23)	*U* = 404	2070 (27 - 37760; 58)	2696 (23 - 36449; 74)	*U* = 2331	*U* = 7096 ***
**Naive CD19$^+$ PNA (MFI)**	990 (192 - 3002; 36)	1650 (482 - 2803; 23)	*U* = 523	1867 (160 - 4984; 57)	1800 (226 - 3861; 76)	*U* = 2029	*U* = 2840 ***
**Naive CD19$^+$ MHC II (MFI)**	35027 (253 - 44118; 37)	31581 (477 - 43729; 23)	*U* = 341	2983 (-69 - 39714; 57)	2165 (7 - 27737; 76)	*U* = 2047	*U* = 7336 **
**Memory CD19$^+$ PNA (MFI)**	959 (44 - 4265; 37)	2185 (384 - 3855; 23)	*U* = 523	3535 (208 - 9370; 57)	3650 (308 - 9958; 75)	*U* = 2059	*U* = 1709 ***
**Memory CD19$^+$ MHC II (MFI)**	21559 (93 - 3194; 370	24639 (196 - 33369; 23)	*U* = 502	2075 (-108 - 36449; 57)	1603 (41 - 26307; 75)	*U* = 1958	*U* = 6838 ***
**Germinal centre CD19$^+$ PNA (MFI)**	966 (166 - 32804; 37)	6270 (411 - 30722; 23)	*U* = 572 *	20810 (213 - 54595; 57)	14837 (294 - 59062; 75)	*U* = 1745	*H* = 50.3 ***, LF < WF***, LF < WM***, LM < WF***, LM < WM*
**Germinal centre CD19$^+$ MHC II (MFI)**	9894 (40 - 67858; 37)	18819 (208 - 7134; 23)	*U* = 566 *	2409 (-108 - 59478; 56)	2798 (1 - 39530; 75)	*U* = 1849	*H* = 27.9 ***, LF > WM**, LM > WF**, LM > WM***
**CD3$^+$: CD19$^+$ ratio**	0.66 (0.36 - 4.00; 37)	0.72 (0.45 - 2.55; 23)	*U* = 503	1.06 (0.50 - 2.85; 48)	1.03 (0.39 - 2.37; 66)	*U* = 1551	*U* = 1843***
**[1]CD11c$^+$ (%) Dendritic Cells**	2.7 (0.7 - 12.0; 40)	3.3 (1.5 - 16.9; 24)	*U* = 662 *	2.1 (0.7 – 9.3; 53)	2.1 (0.6 - 9.3; 73)	*U* = 1771	*H* = 16.0***, LM > WF***, LM > WM**

**CD11c^{+} MHC II (MFI)**	1472 (24 - 23837; 39)	1207 (648 - 3936; 22)	*U* = 379	2395 (-79.2 – 26813; 51)	782 (17.3 - 34110; 70)	*U* = 1530	*U* = 3727
**[1]CD11b^{+} myeloid cells**	5.25 (0.1 - 10.2; 37)	5.91 (2.5 - 13.3; 24)	*U* = 580 *	8.06 (0.3 – 20.9; 50)	8.37 (0.3 - 30.9; 68)	*U* = 1662	*H* = 26.6 ***, LF < WF***, LF < WM***
**[6]M1: Tissue resident macrophages**	17.40 (6.65 - 43.10; 36)	15.90 (5.53 - 33.40; 23)	*U* = 409	7.97 (2.39 – 39.40; 49)	8.26 (1.80 - 29.2; 67)	*U* = 1700	*U* = 5733 ***
**[6]M2: Monocytes**	18.05 (0.52 - 32.50; 36)	18.50 (0.68 - 25.70; 23)	*U* = 407	16.00 (0.30 – 32.90; 49)	14.80 (0.30 - 33.20; 67)	*U* = 1606	*U* = 4141 *
**[6]M3: Hypergranulocytic myeloid cells**	0.65 (0.00 - 3.07; 36)	0.37 (0.00 - 2.71; 23)	*U* = 291	3.94 (0.00 – 28.90; 49)	2.11 (0.00 - 27.20; 67)	*U* = 1285 *	*H* = 49.0 ***, LF < WF***, LF < WM***, LM < WF***, LM < WM***
**[6]M4: PMN**	4.36 (0.00 - 20.70; 36)	5.68 (0.00 - 29.20; 23)	*U* = 420	16.00 (0.00 – 46.80; 49)	16.4 (0.00 - 42.8; 67)	*U* = 1738	*U* = 1051 ***

Supplementary Table 2. Characterisation of lymphocyte populations of wild mice and their comparison to laboratory mice. The table shows the lymphocyte populations for wild and laboratory mice, shown as medians (range; n). Statistical results show comparisons between sex within animal source (*i.e.* laboratory or wild mice), and between animal source. Where there exists a significant difference between sex, mice were grouped by sex and animal source as: laboratory female, laboratory male, wild female and wild male (LF, LM, WF, WM, respectively), with Kruskal-Wallis H-tests (and post-hoc, Dunn-Bonferonni, pairwise comparisons) conducted with significant differences reported. All non-normal data were analysed by Mann-Whitney U-tests, unless stated otherwise. Asterisks denote significant differences as * $P < 0.05$, ** $P < 0.01$, *** $P < 0.001$. Superscripts define cell populations as a percentage of: [1]spleen cells; [2]CD3^{+} cells; [3]CD4^{+} cells; [4]CD8^{+} cells; [5]B cells; [6]CD11b^{+} myeloid cells.

	Prevalence %, median intensity[1], (n)		Overall prevalence, median intensity[1], (n)
	Female Mice	Male Mice	
**Nematode (*Syphacia* sp.)**[2]	88, 42, (81)	93, 51, (100)	91, 40, (181)
**Mites (*Mycoptes musculinus*)**[3]	77, 300, (81)	86, 400, (100)	82, 200, (181)
**Noro**[4]	41 (70)	34 (83)	37 (153)
**Minute**[4]	21 (70)	22 (83)	22 (153)
**Parvo**[4]	89 (70)	94 (83)	92 (153)
**Mouse hepatitis**[4]	30 (70)	29 (83)	29 (153)
**Sendai**[4]	63 (70)	67 (85)	65 (155)
Mycoplasma pulmonis[4]	64 (70)	59 (85)	61 (155)

Supplementary Table 3. Nematode, mite and microbial infections in wild mice. Results are for the HW sample site.

[1]Median intensity for nematode and mite infection only.

[2]No significant effect of sex on the intensity of nematode infection (U = 4286, n_{female} = 81, n_{male} = 100, P = 0.501).

[3]No significant effect of sex on the intensity of mite infection (U = 4457, n_{female} = 81, n_{male} = 100, P = 0.239).

[4]No significant effect of sex on the number of detected microbial infections (U = 2819, n_{female} = 70, n_{male} = 83, P = 0.749).

	Wild			Laboratory		
	**Mean**	**Variance**	**Variance / mean**	**Mean**	**Variance**	**Variance / mean**
**IgG (µg/mL)**	8713.0	51146351.3	5870.1	904.8	4831155.9	5339.3
**IgE (µg/mL)**	132.7	16682.2	125.7	0.99	7.4	7.4
**IgA (µg/g)**	275.3	47024.5	170.8	231.0	5574.2	24.1
**Haptoglobin (µg/mL)**	36.3	14586.6	402.0	18.9	4508.6	237.8
**SAP (µg/mL)**	1.9	1.64	0.85	1.2	0.28	0.23
**AAT (µg/mL)**	252.2	26829.6	106.4	241.3	16528.8	68.5

Supplementary Table 4. Serum protein concentrations in wild and laboratory mice. The mean, variance and variance / mean ratio of serum protein concentrations. Results are for the HW sample site.

Antigen	Cytokine	Laboratory	Wild	Lab. *vs.* Wild
**RPMI**	IL-1β	4.35 ± 15.44, 0.001 (0.001 – 66.16); 19	4.03 ± 12.56, 0.001 (0.001 – 72.490); 70	*U* = 712.5, *P* = 0.466
	IL-4	0.29 ± 0.53, 0.001 (0.001 – 1.860); 19	0.24 ± 0.29, 0.001 (0.001 – 0.840); 70	*U* = 703.0, *P* = 0.673
	IL-6	1.87 ± 3.71, 0.600 (0.001 – 15.910); 19	5.32 ± 13.10, 0.730 (0.001 – 82.370); 70	*U* = 741.5, *P* = 0.443
	IL-10	1.35 ± 3.68, 0.001 (0.001 – 14.650); 19	0.21 ± 0.88, 0.001 (0.001 – 4.790); 70	*U* = 544.0, *P* = 0.034*
	IL-12p40	10.66 ± 11.92, 4.910 (0.001 – 37.270); 19	2.90 ± 3.74, 1.445 (0.001 – 14.940); 70	*U* = 348.0, *P* < 0.001***
	IL-12p70	13.70 ± 20.75, 0.001 (0.001 – 79.050); 19	1.52 ± 6.42, 0.001 (0.001 – 37.890); 70	*U* = 427.5, *P* < 0.001***
	IL-13	2.54 ± 5.67, 0.001 (0.001 – 16.020); 19	0.43 ± 2.23, 0.001 (0.001 – 16.020); 70	*U* = 552.5, *P* = 0.016*
	IFN-γ	1.94 ± 4.26, 0.001 (0.001 – 15.830); 19	2.06 ± 12.52, 0.001 (0.001 – 104.550); 70	*U* = 699.0, *P* = 0.689
	MIP-2α	51.43 ± 103.63, 12.930 (0.150 – 435.360); 19	185.45 ± 446.31, 33.910 (0.340 – 3077.710); 70	*U* = 848.5, *P* = 0.066
**Anti-CD3 / Anti-CD28**	IL-1β	5.02 ± 16.91, 0.001 (0.001 – 71.87); 19	7.71 ± 17.38, 0.001 (0.001 – 84.310); 71	*U* = 789.0, *P* = 0.182
	IL-4	9.27 ± 13.74, 2.51 (0.001 – 44.65); 19	70.72 ± 85.28, 39.750 (0.001 – 457.790); 71	*U* = 1169.5, *P* < 0.001***
	IL-6	19.86 ± 26.92, 7.040 (0.001 – 105.670); 19	32.01 ± 62.06, 14.000 (0.001 – 343.930); 71	*U* = 794.0, *P* = 0.237
	IL-10	4.78 ± 7.08, 0.001 (0.001 – 22.750); 19	6.16 ± 14.31, 1.690 (0.001 – 109.810); 71	*U* = 777.0, *P* = 0.301
	IL-12p40	13.22 ± 12.27, 8.390 (0.001 – 46.460); 19	3.90 ± 3.71, 2.920 (0.001 – 15.400); 71	*U* = 297.5, *P* < 0.001***
	IL-12p70	21.32 ± 27.28, 0.001 (0.001 – 65.840); 19	3.66 ± 11.79, 0.001 (0.001 – 70.480); 71	*U* = 556.0, *P* = 0.182
	IL-13	16.85 ± 26.42, 0.001 (0.001 – 88.200); 19	5.75 ± 22.93, 0.001 (0.001 – 187.540); 71	*U* = 541.0, *P* = 0.119
	IFN-γ	64.20 ± 118.05, 9.490 (0.001 – 456.100); 19	188.52 ± 277.00, 71.090 (0.001 – 1687.320); 71	*U* = 966.0, *P* = 0.004**
	MIP-2α	70.86 ± 91.07, 35.75 (0.320 – 392.61); 19	288.25 ± 683.68, 114.700 (1.480 – 5471.880); 71	*U* = 1008.0, *P* < 0.001***
**CpG**	IL-1β	41.77 ± 53.01, 0.240 (0.001 – 135.10); 19	5.71 ± 13.41, 0.001 (0.001 – 53.77); 70	*U* = 445.5, *P* = 0.013*
	IL-4	1.92 ± 2.21, 0.440 (0.001 – 5.650); 19	0.62 ± 1.22, 0.330 (0.001 – 7.110); 70	*U* = 521.0, *P* = 0.127
	IL-6	496.41 ± 519.76, 292.530 (2.630 – 1843.080); 19	294.52 ± 276.46, 228.900 (1.570 – 1440.260); 70	*U* = 546.0, *P* = 0.233
	IL-10	73.41 ± 90.66, 28.770 (0.001 – 267.330); 19	45.01 ± 49.96, 34.180 (0.001 – 377.240); 70	*U* = 664.0, *P* = 0.992
	IL-12p40	378.89 ± 341.09, 236.750 (10.300 – 1200.880); 19	197.30 ± 165.26, 163.850 (4.410 – 679.13); 70	*U* = 423.0, *P* = 0.015*
	IL-12p70	68.14 ± 83.71, 0.790 (0.001 – 211.140); 19	9.69 ± 26.93, 0.056 (0.001 – 143.900); 70	*U* = 506.0, *P* = 0.091
	IL-13	37.58 ± 46.34, 1.490 (0.001 – 110.230); 19	4.39 ± 15.19, 0.001 (0.001 – 81.860); 70	*U* = 386.0, *P* < 0.001***
	IFN-γ	15.10 ± 31.53, 3.140 (0.001 – 131.780); 19	13.28 ± 32.98, 1.040 (0.001 – 197.050); 70	*U* = 644.0, *P* = 0.833
	MIP-2α	111.71 ± 81.10, 107.00 (5.540 – 304.830); 19	172.26 ± 687.90, 44.055 (1.230 – 5716.820); 70	*U*= 460, *P* = 0.040*
**LPS**	IL-1β	6.73 ± 18.12, 0.001 (0.001 – 66.98); 19	4.75 ± 12.36, 0.001 (0.001 – 78.270); 70	*U* = 684.0 *P* = 0.823
	IL-4	0.87 ± 0.94, 0.47 (0.001 – 2.400); 19	0.34 ± 0.43, 0.230 (0.001 – 2.480); 70	*U* = 508.0, *P* = 0.98
	IL-6	26.41 ± 22.31, 20.480 (0.001 – 68.660); 19	20.51 ± 21.26, 13.500 (0.001 – 97.050); 70	*U* = 571.0, *P* = 0.347
	IL-10	20.18 ± 27.00, 2.060 (0.001 – 81.400); 19	6.44 ± 11.54, 3.630 (0.001 – 81.620); 70	*U* = 5805, *P* = 0.391
	IL-12p40	15.09 ± 14.52, 9.950 (0.130 – 51.220); 19	3.72 ± 2.78, 3.355 (0.001 – 12.190); 70	*U* = 2980, *P* < 0.001***
	IL-12p70	22.02 ± 28.12, 0.001 (0.001 – 82.620); 19	1.53 ± 6.69, 0.001 (0.001 – 36.860); 70	*U* = 418.5, *P* < 0.001***
	IL-13	11.93 ± 14.79, 0.001 (0.001 – 34.130); 19	1.10 ± 3.97, 0.001 (0.001 – 21.980); 70	*U* = 430.0, *P* < 0.001***
	IFN-γ	3.36 ± 4.14, 0.230 (0.001 – 13.710); 19	3.65 ± 11.32, 0.570 (0.001 – 87.350); 70	*U* = 634.0, *P* = 0.753
	MIP-2α	143.03 ± 109.85, 111.58 (2.810 – 352.550); 19	185.53 ± 271.40, 112.510 (0.030 – 2068.290); 70	*U* = 679.0, *P* = 0.889

Peptidoglycan	IL-1β	46.84 ± 58.00, 9.020 (0.001 – 161.21); 19	27.11 ± 28.99, 21.64 (0.001 – 150.790); 70	U = 680.0, P = 0.880
	IL-4	2.07 ± 2.40, 0.430 (0.001 – 5.920); 19	0.86 ± 1.24, 0.535 (0.001 – 5.950); 70	U = 577.0, P = 0.362
	IL-6	76.94 ± 98.54, 27.050 (0.001 – 285.560); 19	62.47 ± 74.21, 39.595 (0.450 – 349.730); 70	U = 680, P = 0.881
	IL-10	260.72 ± 286.37, 128.360 (42.550 – 1060.450); 19	180.13 ± 146.20, 140.725 (34.730 – 1032.360); 70	U = 665.0, P = 1.000
	IL-12p40	192.78 ± 294.21, 48.350 (11.340 – 1040.81); 19	52.05 ± 62.80, 33.420 (11.200 – 333.100); 70	U = 447.0, P = 0.029*
	IL-12p70	97.53 ± 129.35, 9.610 (0.001 – 400.780); 19	13.39 ± 26.42, 5.235 (0.001 – 139.930); 70	U = 499.0, P = 0.096
	IL-13	32.28 ± 40.53, 0.001 (0.001 – 101.430); 19	3.28 ± 11.78, 0.001 (0.001 – 65.060); 70	U = 420.0, P < 0.001***
	IFN-γ	47.98 ± 131.07, 1.140 (0.001 – 535.730); 19	16.83 ± 43.93, 1.630 (0.001 – 237.770); 70	U = 656.0, P = 0.928
	MIP-2α	15559.44 ± 1674.56, (953.64 (9.0 – 5534.0); 19	2370.94 ± 4996.54, 999.92 (10.000 – 38832.000); 70	U = 730.0, P = 0.515

Supplementary Table 5. Statistical analyses of cytokine data. Values are: mean ± S.D., median, (range); sample size. Statistics are Mann-Whitney U-tests. Asterisks denote significant differences (**P* < 0.05, ***P* < 0.01, ****P* < 0.001). Results are for the HW sample site.

Antibody	Fluorochrome	Clone	Source	Panel
Fixable Live/Dead	Aqua	N/A	N/A	A-H
Anti-CD3e	PE-Cy7	145-2C11	Armenian Hamster	A, C
Anti-CD4	APC-H7	GK1.5	Rat	A, C
Anti-CD8a	PerCP-Cy5.5	53-6.7	Rat	A, C
Anti-CD11a	eFluor450	M17/4	Rat	A, C
Anti-CD49d	FITC	R1-2	Rat	A
Anti-CD44	APC	IM7	Rat	A
Anti-CD62L	PE	MEL-14	Rat	A
Anti-CD25	APC	PC61.5	Rat	C
Anti-FoxP3	FITC	FJK-16s	Rat	C
Anti-KLRG1	PE	2F1	*Mesocricetus auratus*	C
Anti-CD3e	APC-eFluor780/ eFluor450	145-2C11	Armenian Hamster	D, F
Anti-NKp46	eFluor450/ PE	29A1.4	Rat	D, F
Anti-CD11b	FITC	M1/70	Rat	D
Anti-CD27	PE/ APC	LG.7F9	Armenian Hamster	D
Anti-CD11b	APC-eFluor780	M1/70	Armenian Hamster	D
Anti-CD69	PerCP-Cy5.5	H1.2F3	*Mesocricetus auratus*	D
Anti-KLRG1	PE-Cy7	2F1	Rat	D
Anti-CD62L	APC-eFluor780	MEL-14	Rat	F
Anti-Ly49D	APC	4E5	Rat	F
Anti-Ly49G2	PerCP-eFluor710	4D11	Mouse	F
Anti-Ly49H	FITC	3D10	Rat	F
Anti-NKG2D	PE-Cy7	CX5	Rat	F
Anti-CD19	APC-eFluor780	1D3	Rat	G
Anti-CD38	APC	90	Rat	G
Anti-GL7	eFluor450	GL-7	Rat	G
Anti-IgD	PE	11-26c	Rat	G
Peanut Agglutin (PNA)	FITC	N/A	N/A	G
Anti-MHC-II	PerCP-eFluor710	M5/114.15.2	Rat	G, H
Anti-CD3e	PE-Cy7	145-2C11	Rat	H
Anti-CD11c	FITC	N418	Armenian Hamster	H
Anti-F4/80	APC	BM8	Rat	H
Anti-Ly6G	eFluor450	RB6-8C5	Rat	H

Supplementary Table 6. Antibodies, fluorochromes and staining panels used in the flow cytometry analyses. Compensation control beads were from BD Biosciences (Oxford, UK). Intracellular staining with anti-FoxP3 was performed following permeabilization of cells using the permeabilization buffer from the e-Bioscience (Hartfield, UK) FoxP3 staining set.
